# Omental macrophages secrete chemokine ligands that promote ovarian cancer colonization of the omentum via CCR1

**DOI:** 10.1038/s42003-020-01246-z

**Published:** 2020-09-22

**Authors:** Venkatesh Krishnan, Supreeti Tallapragada, Bruce Schaar, Kalika Kamat, Anita M. Chanana, Yue Zhang, Sonia Patel, Vinita Parkash, Carrie Rinker-Schaeffer, Ann K. Folkins, Erinn B. Rankin, Oliver Dorigo

**Affiliations:** 1 Department of Obstetrics and Gynecology, Division of Gynecologic Oncology, Stanford School of Medicine, Stanford Cancer Institute, Stanford, CA USA; 2 Genome Technology Center, Stanford, CA USA; 3 Department of Obstetrics and Gynecology, David Geffen School of Medicine, University of California, Los Angeles, CA USA; 4 Department of Physiology and Chemistry, Genentech, San Francisco, CA USA; 5 Department of Thoracic Head and Neck Medical Oncology, Division of Cancer Medicine, University of Texas Health Science Center at Houston, Houston, TX USA; 6 Department of Pathology, Yale School of Medicine and Yale School of Public Health, New Haven, CT USA; 7 Department of Surgery, Division of Urology, University of Chicago, Chicago, IL USA; 8 Department of Pathology, Stanford School of Medicine, Stanford, CA USA; 9 Department of Radiation Oncology, Stanford School of Medicine, Stanford, CA USA; 10 Stanford Women’s Cancer Center, Stanford, CA USA

**Keywords:** Ovarian cancer, Phagocytes, Chemokines

## Abstract

The omentum is the most common site of ovarian cancer metastasis. Immune cell clusters called milky spots are found throughout the omentum. It is however unknown if these immune cells contribute to ovarian cancer metastasis. Here we report that omental macrophages promote the migration and colonization of ovarian cancer cells to the omentum through the secretion of chemokine ligands that interact with chemokine receptor 1 (CCR1). We found that depletion of macrophages reduces ovarian cancer colonization of the omentum. RNA-sequencing of macrophages isolated from mouse omentum and mesenteric adipose tissue revealed a specific enrichment of chemokine ligand CCL6 in omental macrophages. CCL6 and the human homolog CCL23 were both necessary and sufficient to promote ovarian cancer migration by activating ERK1/2 and PI3K pathways. Importantly, inhibition of CCR1 reduced ovarian cancer colonization. These findings demonstrate a critical mechanism of omental macrophage induced colonization by ovarian cancer cells via CCR1 signaling.

## Abstract (web summary)

Krishnan et al. find that CCR1 ligands CCL6 and CCL23 secreted by murine and human macrophages, respectively, enhance metastatic colonization of ovarian cancer cells to the omentum in manner dependent on chemokine receptor 1 (CCR1). This study suggests that targeting CCR1 or CCL23 in ovarian cancer may be a therapeutic strategy.

## Introduction

Ovarian cancer is the seventh most common cancer in women with 300,000 new cases and 185,000 deaths worldwide in 2018^1^. In 2020, an estimated 21,750 new cases of ovarian cancer will be diagnosed in the USA and 13,940 women will die from the disease^2^. Ovarian cancer originates in the ovary or fallopian tube and metastasizes throughout the peritoneal cavity^3^. Metastatic disease, rather than the primary tumor, is the cause of ovarian cancer-related deaths^4^. However, the fundamental mechanisms that support intraperitoneal metastasis are not well understood.

The omentum is a preferred metastatic site for ovarian cancer cells. The majority of women diagnosed with high grade serous ovarian cancer present with omental metastases^5–7^. In experimental models of ovarian cancer, ovarian cancer cells metastasize to the omentum within 1–6 h of injection^8^. The omentum is an adipose tissue that contains secondary lymphoid structures with a variety of cell types, including adipocytes, blood vessels, and clusters of leukocytes known as milky spots^9–15^. Milky spots contain macrophages, B, T, and NK cells, which rapidly efflux into the peritoneum during pathogenic challenge^10,16^. While various cell types, including neutrophils, mesothelial cells, and adipocytes, have been shown to support ovarian cancer metastasis to the omentum, little is known regarding the role of tissue-resident immune cells in omental metastasis^7,15,17^.

Macrophages enhance metastasis in several cancer models. In breast cancer, macrophages facilitate the early hematogenous dissemination of cancer cells from the primary tumor by altering vascular permeability and facilitating migration into the bloodstream^18^. Chemokines enhance the interactions between cancer cells and macrophages in a chemokine cascade that promotes breast cancer metastasis^19^. In ovarian cancer, macrophage-driven inflammation can promote intraperitoneal metastasis and ascites production^20^. Specific subsets of omental macrophages might be involved in promoting metastasis to the omentum^17^.

In this study, we describe the role of omental macrophage-derived chemokine ligands in promoting ovarian cancer cell colonization of the omentum. Gene expression profiling of mouse omental macrophages compared to mesenteric adipose macrophages shows distinct gene expression patterns with significant upregulation of the chemokine ligand CCL6 prior to and during colonization of the omentum by ovarian cancer cells. CCL6 induces the migration of ovarian cancer cells as an important step in colonization via the chemokine receptor CCR1. Furthermore, inactivation of CCR1 in mouse ovarian cancer cells blocks CLL6 induced migration and abolishes their ability to colonize the omentum. In ovarian cancer patients, the expression of CCR1 is associated with a poorer prognosis. In addition, CCL23, the human homolog of CCL6, is expressed on macrophages isolated from human omentum. These observations validate the importance of CCR1 and its ligand CCL23 in human ovarian cancer.

## Results

### Omentum as a niche for ovarian cancer metastasis

**Fig. 1:**
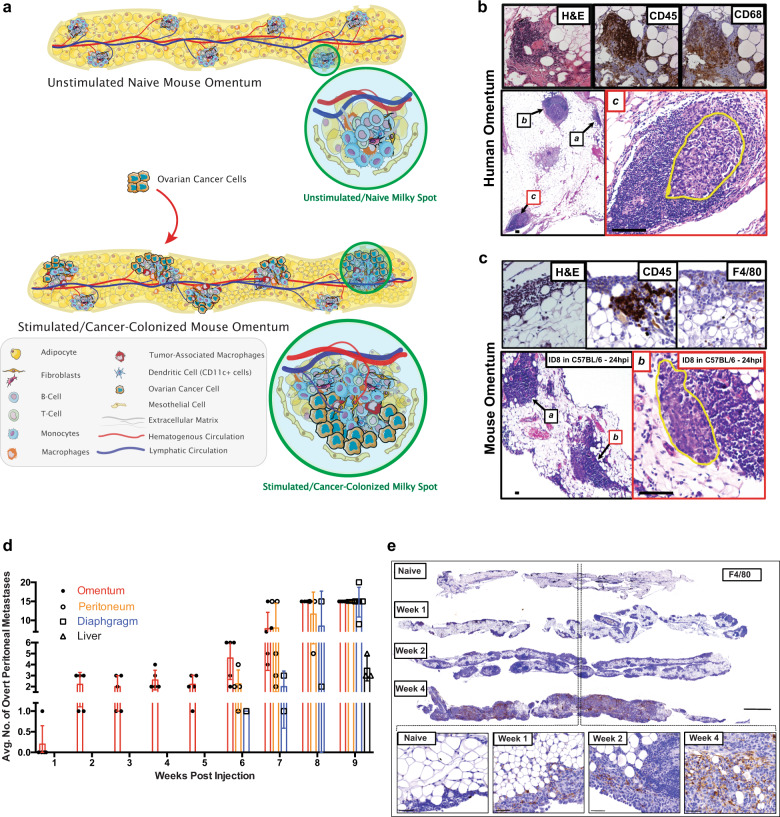
Omentum as a niche for ovarian cancer metastasis. **a** Representation of a naïve unstimulated milky spot and ovarian cancer metastasis activated milky spots within the mouse omentum. The number of macrophages associated with the expanding cancer-immune cells regions increase and cancer cells grow past milky spots into the central adipose region, eventually replacing it. **b**, **c** Omental milky spots can be detected in human and mouse samples. IHC of naive omental tissue sections stained for H&E, CD45 and macrophages (mouse F4/80 and human CD68). Uninvolved milky spot in human (**b**; inset **a**, **b**) and mouse (**c**; inset **a**). Microscopic ovarian metastases were identified within milky spots in human omenta (**b**; inset **c**), and mouse (**c**; inset **b**); scale bar = 100 µm. **d** C57BL/6 mice were injected with ID8 cancer cells and overt (>1 mm) peritoneal metastases over a period of 9 weeks were quantitated for metastatic spread within the abdomen (*n* = 6). **e** Representative digital scans of whole mouse omental sections stained for F4/80. Milky spots at week 0 (naive un-injected omentum) and week 1, week 2, week 3, week 4, post injection of ID8 cells. (Bottom) ×40 magnification of milky spot regions at each time point. (Scale bar = 1×, 1000 μM; 40×, 50 μM).

The omental vasculature is characterized by the presence of numerous branching blood vessels ending in glomerulus-like capillary beds near the periphery of the omentum. Immune cells aggregate around and within these capillary beds to form milky spots. The milky spots contain clusters of monocytes (CD11b^+^), B cells, macrophages, CD11c^+^ cells, and T cells all under a gap in the mesothelial lining (Fig. 1a). The endothelial lining of the capillaries and the overlying mesothelium are adapted to facilitate the transmigration of immune cells. Additional structural elements are provided by, fibroblasts, mesenchymal cells, collagen, and elastic fibers (Fig. 1a).

To investigate the role of tissue-resident macrophages in metastatic colonization to the omentum, we first verified the presence of macrophages in milky spots in naive human and murine omentum (Fig. 1b, c). Interestingly, in omenta from ovarian cancer patients without gross disease, microscopic metastases were restricted to milky spots and were not observed in the adipose tissue at this early stage (Fig. 1b). Similarly, microscopic metastases in murine omentum were localized and confined to the milky spots at early timepoints (24 h) after intraperitoneal (i.p.) injection of ID8 cells (Fig. 1c).

We next studied the dynamics of intraperitoneal metastasis in our murine ovarian cancer model. ID8 murine ovarian cancer cells were injected i.p. into C57BL/6 mice and the anatomic distribution of metastatic colonization was followed over time. Interestingly, the omentum was the first site of macroscopic metastases within one week of injection and remained the only site of peritoneal metastasis for 6 weeks after which metastases appeared at other sites (Fig. 1d). This is similar to observations in patients with ovarian cancer who frequently present with omental metastasis upon diagnosis but may not have other sites of significant disease burden^5,6^. Milky spots increased in number and size after cancer cell colonization at week 1 post injection. The number of immune cells, in particular macrophages, was found to increase by week 2. We observed continued expansion of tumor from milky spots into the adipose tissue of the omentum following early colonization (Fig. 1e). These findings suggest that the immune cell-rich milky spots are sites of early metastasis of ovarian cancer cells within the omentum.

### Macrophages promote ovarian cancer colonization of the omentum

**Fig. 2:**
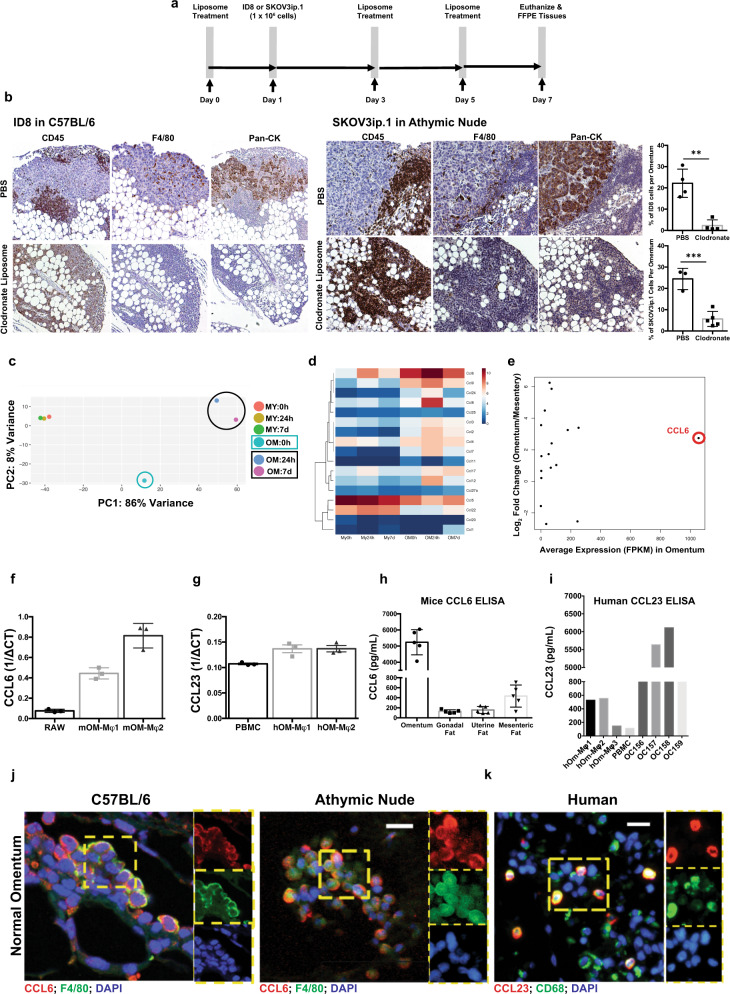
Ovarian cancer cell colonization of the omentum is macrophage dependent. **a** Experimental design of clodronate liposome treatment for 7 dpi of ID8/SKOV3ip.1 in vivo metastasis assay. **b** Representative omental images of IHC for lymphocytes (CD45), macrophages (F4/80) and cancer cells (pan-CK). Quantification of DAB (pan-CK) staining area for each model is shown on the right (*p < 0.05; *n* = 3). (**c**–**e**) **c** Principal-component analysis (PCA) of genes expressed in the omental (OM) and mesenteric (MY) adipose tissue macrophages. Each data point represents macrophages derived from three omenta (*n* = 3). **d** Heat map of all chemokines expressed in (**c**). **e** Plot of chemokine genes highly upregulated in naive omental macrophages compared to naive mesenteric macrophages (timepoint zero). **f** Gene expression analysis of CCL6 on mouse naive omental macrophages isolated from C57BL/6 mice (*n* = 2) by q-RT PCR. RAW 264.7 cells were used as positive control. **g** Gene expression analysis of CCL23 on human omental macrophages isolated from patient with benign disease (*n* = 2). PBMCs were used as positive control. **h** ELISA of CCL6 levels in conditioned media generated from weight matched naive omentum and peritoneal adipose tissues (*n* = 3). **i** ELISA of CCL23 levels on conditioned media generated from benign patient samples. Omental macrophages (hOM-Mϕ1, 2, and 3) and whole-omental-tissue (OC157, 158, and 159) each represent a different benign sample. Conditioned media derived from PBMCs were used as control. **j** Immunofluorescence staining for co-localization of CCL6 (red) and F4/80 (green) and nuclei (blue, DAPI) on naive omental tissue from C57BL/6 and athymic nude mice. **k** Similarly, CCL23 (red) and CD68 (green) on the human omentum derived from benign patient samples. (scale bar = 20 µm).

Previous studies have demonstrated in both murine (ID8) and human ovarian cancer models (CaOV3, HeyA8, or SKOV3ip.1) that colonization of the omentum was independent of B-, T-, and NK- cells^21^. To determine the role of macrophages in omental colonization, we depleted macrophages in C57BL/6 and athymic nude mice using clodronate liposomes prior to i.p. injection of mouse ID8 or human SKOV3ip.1 ovarian cancer cells, respectively (Fig. 2a). Strikingly, we observed an 11-fold reduction in the ovarian cancer burden in the omentum of macrophage depleted mice at 7 days following i.p. injection of tumor cells (Fig. 2b). Interestingly, when macrophages were depleted using clodronate liposome three days after injection of SKOV3ip.1 cells, we failed to observe a decrease in omental colonization. (Supplementary Fig. 1). These findings support our hypothesis that macrophages are crucial for the initiation of ovarian cancer colonization of the omentum.

### Omental macrophage-derived conditioned media promote migration of ovarian cancer cells

Our findings above indicate an important role for omental macrophages in metastatic colonization of ovarian cancer. Therefore, we investigated whether omental macrophages can induce migration of ovarian cancer cells as an important aspect of metastatic colonization. For this purpose, CD11b^+^ cells were isolated from normal mouse omenta and cultured to generate conditioned media (Supplementary Fig. 2a). CD11b^+^ cells isolated from murine gonadal fat were used for comparison since our prior studies had demonstrated a lack of early metastasis to intraperitoneal adipose outside of the omentum^21^. Conditioned media generated by CD11b^+^ cells isolated from mouse omenta significantly increased migration of ID8 ovarian cancer cells (Supplementary Fig. 2b). Compared to conditioned media from omental CD11b^+^ cells, conditioned media from gonadal fat CD11b^+^ cells had only minor effects on ovarian cancer cell migration. To validate these findings in human tissue, we isolated macrophages from human omentum collected from individuals with benign disease (CD45^+^CD14^+^CD68^+^ cells; Supplementary Fig. 3b). Similar to the effects shown in murine CD11b^+^ cells, all human macrophage conditioned media significantly enhanced the migration of human SKOV3.ip1 cells (Supplementary Fig. 2d). These findings suggest that omental macrophages secrete chemoattractants into the tumor microenvironment that enhance migration and, therefore, the metastatic potential of ovarian cancer cells.

### RNA-sequencing of omental macrophage reveals distinct gene expression patterns

We next investigated the mechanisms that regulate the omental macrophage-induced colonization of the omentum. For this purpose, changes in gene expression were studied in macrophages during the omental colonization process. Macrophages (CD45^+^/CD11b^+^/F4/80^+^) were isolated from mouse omenta prior to injection of ID8 cells, and 24 hours and seven days post-injection (dpi). RNA sequencing analysis was performed on macrophages at the respective time points (Supplementary Fig. 3a). RNA isolated from mesenteric adipose tissue macrophages was used for comparison. Hierarchical clustering and principal-component analysis (PCA) of 12756 genes expressed by omental macrophages during intraperitoneal metastasis resulted in various important observations. First, naive omental macrophages showed a gene signature distinct from mesenteric adipose tissue-derived macrophages before injection of ID8 cells (1283 genes were differentially expressed between omentum and mesentery derived macrophages). Second, omental macrophages showed a significant change in gene expression after the injection of ID8 cells, while there was no change in the gene signature from mesenteric adipose tissue-derived macrophages. (Fig. 2c). Genes expressed in omental macrophages at 24 h and 7 dpi were significantly enriched for cell adhesion, immune response, cytokine-cytokine receptor interaction, and chemotaxis (Supplementary Fig. 3c). Among the chemokines, CCL6 was found to be the gene with the highest fold upregulation in naive omental macrophages compared to naive mesenteric macrophages (timepoint zero). In addition, we observed a significant increase of CCL6 expression in omental but not mesenteric macrophages during colonization (Fig. 2d, e). Since CCL6 is expressed primarily by macrophages^22–24^, we hypothesized that macrophage-derived CCL6 might promote early omental metastasis by interaction with its receptor CCR1 expressed on ovarian cancer cells.

We validated the expression of CCL6 and CCL23 in flow-sorted mouse or human omental macrophages, respectively by qRT-PCR (Fig. 2f, g). Conditioned media generated from naive mouse omenta contained high levels of secreted CCL6 (Fig. 2h). High levels of CCL23 were also detected in conditioned media from whole human omentum without tumor and human omentum derived macrophages (Fig. 2i). Immunofluorescence staining of tissue sections further confirmed the presence of CCL6-expressing F4/80^+^ macrophages in mouse omenta and CCL23-expressing CD68^+^ macrophages in human omenta (Fig. 2j, k).

### CCL6 and CCL23 promote ovarian cancer migration

**Fig. 3:**
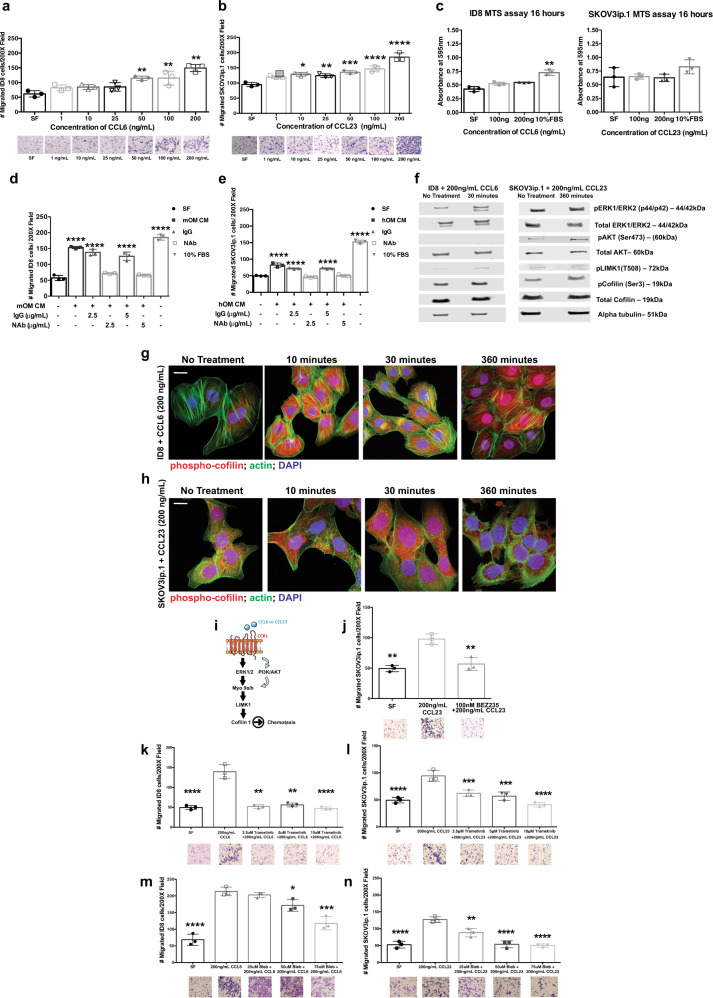
Ovarian cancer cells show enhanced migration toward CCL6/CCL23 by activating Myosin9 via the Cofilin signaling in vitro. **a**, **b** Transwell migration assay of ID8/SKOV3ip.1 cells toward varying concentrations of CCL6 and CCL23 with representative crystal violet staining. Statistical significance (*p < 0.05) was determined for all conditions versus the serum-free media control by ordinary one-way ANOVA analysis. **c** Quantitation of cell proliferation of ID8/SKOV3ip.1 cells in the presence of CCL6/CCL23 (100 ng/mL–200 ng/mL) by MTS assay. 10% FBS was used as the positive control. **d**, **e** Transwell migration assays with ID8 cells in the presence of anti-CCL6 (2.5 μg/mL and 5 μg/mL; *n* = 3) toward mouse omental conditioned media and SKOV3ip.1 cells toward human omental conditioned media in the presence of anti-CCL23 (2.5 μg/mL–5 μg/mL; *n* = 3). Statistical significance was determined by two-way ANOVA analysis for each condition corresponding to the serum-free (SF) control (*p < 0.05). **f** Western blot analysis of ID8 treated with CCL6 (200 ng/mL) and SKOV3ip.1 treated with CCL23 (200 ng/mL) (*n* = 3). **g**, **h** Immunofluorescence staining for phospho-cofilin (red), actin cytoskeleton (green), and nuclei (blue, DAPI) on ID8 and SKOV3ip.1 ovarian cancer cells treated with CCL6 or CCL23, respectively, at different time points (*n* = 3); (scale bar = 20 µm). **i** Schematic representation of the CCR1-CCL6/CCL23 signaling pathway leading to increased migratory potential of the ovarian cancer cells. **j** Transwell migration assay of SKOV3ip.1 toward CCL23 treated with BEZ235 (PI3K Inhibitor) (**p < 0.008). **k**, **l** Transwell migration assay of ID8 toward CCL6 and SKOV3ip.1 cells toward CCL23 treated with trametinib (MEK inhibitor). **m**, **n** Transwell migration assay of ID8 toward CCL6 and SKOV3ip.1 cells toward CCL23 treated with blebbistatin (myosin inhibitor). Statistical significance was determined by two-way ANOVA analysis with Tukey’s multiple comparison *t*-test with each condition compared to the 200 ng/mL CCL23 treatment condition.

We hypothesized that CCL6 and CCL23 are important mediators of colonization in our mouse model and in human omentum respectively. Both chemokine ligands can induce migration which is a crucial aspect of colonization. Therefore, we investigated the ability of CCL6 and CCL23 to enhance migration of mouse and human ovarian cancer cell lines. Both murine ID8 and human SKOV3ip.1 cell lines showed a concentration-dependent migration toward CCL6 and CCL23 (Fig. 3a, b). This effect was not due to increased cell proliferation as neither ID8 nor SKOV3ip.1 cells cultured in serum-free medium in the presence of 100 ng/mL CCL6 or 200 ng/mL CCL23 showed differences in cell proliferation (Fig. 3c). Antibody-mediated neutralization of CCL6 abrogated the migration of ID8 cells toward mouse omentum-conditioned media (Fig. 3d). Similarly, induction of SKOV3ip.1 migration toward conditioned media from human omentum was blocked by CCL23 neutralizing antibody (Fig. 3e).

To better understand the cellular mechanisms of CCL6 and CCL23 mediated migration, we performed microarray gene expression analysis on CCL6 treated ID8 cells. We found that CCL6 induced upregulation of 98 genes and downregulation of 108 genes. Upregulated genes were clustered in pathways that regulate cell migration, such as integrin-mediated cell adhesion, MAPK signaling pathway, and signaling by RhoGTPases (Supplementary Table 1). Among important regulators of migration, Myosin 9A (Myo9A), LIMK1, and Cofilin were significantly upregulated in CCL6 treated ID8 cells compared to untreated cells (Supplementary Fig. 4a–c). Phospho-proteomic profiling of CCL6 treated ID8 cells and CCL23 treated SKOV3ip.1 cells showed increased levels of pERK1/2 in both cell lines and increased pAkt in SKOV3ip.1 cells (Supplementary Fig. 4d, e). pERK1/2 and pAkt regulate migration in part by phosphorylating LIMK1 and p-Cofilin^25–28^ (Fig. 3f and Supplementary Fig. 4f–i). We confirmed the upregulation of p-cofilin by immunofluorescence in both ID8 and SKOV3ip.1 as early as 10 min post treatment with CCL6 and CCL23, respectively (Fig. 3g, h). We hypothesized that phosphorylation of ERK1/2 by CCL6 and CCL23 regulates MYO9A, which modulates downstream p-LIMK1 and p-Cofilin in the migration pathway (Fig. 3i). In vitro migration of ID8 and SKOV3ip.1 cells toward CCL6 or CCL23 was inhibited by pharmacologic targeting of MEK (trametinib) and PI3K pathway (BEZ235) respectively (Fig. 3j–l). In addition, inhibition of Myosin-9 (blebbistatin) resulted in a two-fold decreased migration of both ID8 and SKOV3ip.1 cells toward CCL6 and CCL23, respectively (Fig. 3m, n). The concentrations of BEZ235, trametinib and blebbistatin used in the migration assays did not affect the viability of ovarian cancer cells (Supplementary Fig. 4j–n). Our findings suggest that CCL6 or CCL23 induce migration by activating the ERK and PI3-Kinase pathways resulting in enhanced downstream signaling via MYO9A and p-Cofilin.

### Genetic and pharmacological inhibition of CCR1 in human and mouse ovarian cancer cells reduces migration toward CCL6/CCL23

**Fig. 4:**
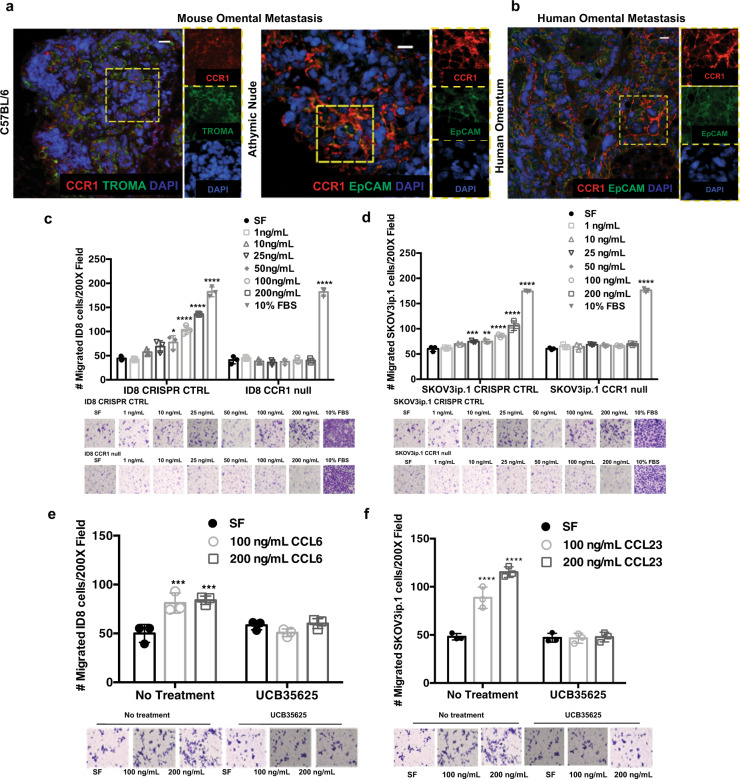
Genomic deletion and pharmacological inhibition of CCR1 in human and mouse ovarian cancer cells inhibits their migratory capacity toward CCL6/CCL23 in vitro. **a** Immunofluorescence for CCR1 (red), cytokeratin (green), and nuclei (blue, DAPI) on ID8 cells metastasized to omentum in C57BL/6 and athymic nude mice (scale bar = 20 µm). **b** Immunofluorescence for CCR1 (red), EPCAM (green), and nuclei (blue, DAPI) on omenta derived from patients with high-grade serous cancer (scale bar = 20 µm). **c**, **d** Transwell migration assay of ID8 and SKOV3ip.1 with genomic deletion of CCR1 toward CCL6 and CCL23. **e**, **f** Transwell migration assay of ID8 and SKOV3ip.1 toward CCL6 and CCL23 with pharmacological inhibition of CCR1 using a small molecule antagonist UCB35625 (100 nM). Data shown represent mean and s.d of technical replicates (*n* = 3) for each condition. Statistical significance was determined by ordinary two-way ANOVA analysis comparing each condition to serum free (SF) media (**p < 0.005; ****p < 0.0001).

CCL6 and CCL23 signal through the CCR1 chemokine receptor. To determine the role of CCR1 in macrophage-induced omental colonization, we first verified the expression of CCR1 in ID8 and SKOV3ip.1 cells (Supplementary Fig. 5a). CCR1 was expressed in ID8 and SKOV3ip.1 derived tumor tissue (Fig. 4a, b). The functional importance of CCR1 in CCL6 and CCL23 induced migration was examined in ID8 and SKOV3ip.1 cells with deletion of *CCR1* by CRISPR-Cas9. Deletion of *CCR1* was verified by sequencing the targeted locus and by qRT-PCR for CCR1 expression (Supplementary Fig. 5b–e). Induction of migration by CCL6 was completely abrogated in ID8-*CCR1*-null cells. (Fig. 4c). Similarly, *CCR1*-deleted SKOV3ip.1 cells failed to respond to CCL23 induced migration (Fig. 4d). No differences in tumor cell proliferation or viability were observed in the *CCR1*-null cell lines relative to the parental cells (Supplementary Fig. 5f, g). We next utilized a small molecule inhibitor of CCR1 (UCB35625) to investigate the therapeutic potential of CCR1 blockade in reducing ovarian cancer migration. CCR1 inhibition by UCB35625 blocked CCL6 and CCL23 induced migration of ID8 and SKOV3ip.1, respectively (Fig. 4e, f).

### *CCR1* knock-down in ovarian cancer cells reduces colonization of the omentum

**Fig. 5:**
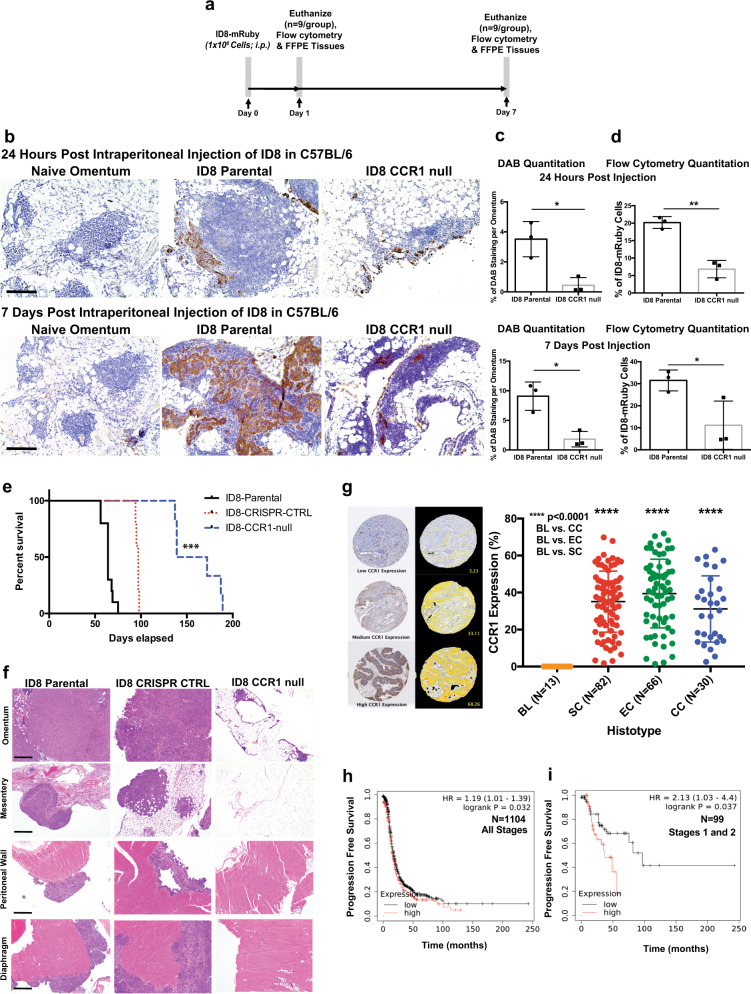
Colonization of the omentum is disrupted by CCR1 knock-down in the cancer cells. **a** Schematic of experimental design for the 24hpi and 7dpi of ID8 in vivo metastasis assay. **b** Representative sections of omenta collected at 24 hpi and 7 dpi of stained for CK8/18 by IHC (scale bar = 100 µm). **c** Quantification of DAB staining area as an indicator of ID8 cancer cell burden in omental tissues. **d** Quantitation of flow cytometry analysis of ID8-mRUBY cells colonizing omentum at 24 hpi and 7 dpi (*p < 0.05). **e** Kaplan–Meier curve showing percent-survival after i.p injection of ID8- parental, CRISPR-Control, or *CCR1*-null cells (**p < 0.001) (*n* = 6/group). **f** Representative H&E stained peritoneal tissues at experimental end point. H&E images of ID8-*CCR1*-null group are from mice euthanized at 97 days, the experimental end point of the ID8-CRISPR-Control group. (scale bar = 100 µm). **g** Examples of 3 patient cores of endometrioid histology from a tissue microarray stained for CCR1 and IHC quantitation of CCR1 levels in a tissue microarray with different ovarian cancer histologies (****p < 0.0001). **h**, **i** Kaplan–Meier survival analysis (log-rank test) of CCR1 expression on progression free survival of serous ovarian cancer patients in **h** all stages and **i** stages I and II.

We next studied if CCR1 is important in omental colonization by ovarian cancer cells in vivo. mRuby-labeled ID8 parental cells and ID8-*CCR1*-null cells were injected i.p. into C57BL/6 mice. At 24 hours post-injection (hpi) and seven dpi, omenta were harvested and assessed for metastasis by histology and flow cytometry (Fig. 5a). Numerous foci of ID8 parental (control) cells were observed within the milky spots of the omentum 24 hpi and seven dpi. At both time points, omental tissue contained significantly less ID8-*CCR1*-null cells compared to ID8-CRISPR-control cells with a three-fold reduction at 24 h and a seven-fold reduction at 7 days post injection (Fig. 5b–d and Supplementary Fig. 6a–f).

We then determined whether CCR1 is associated with a more aggressive in vivo progression of tumor growth and its impact on survival. ID8-parental, ID8-CRISPR-Control, or ID8-*CCR1*-null cells were injected i.p. into mice. Mice injected with ID8-*CCR1*-null cells had a significantly slower progression of tumor growth and longer survival compared to mice injected with ID8-CRISPR-Control or ID8 parental cells (Fig. 5e). After 9 weeks, all mice (*n* = 5/group) injected with ID8-parental cells had developed ascites and extensive peritoneal metastases (Fig. 5f). In contrast, none of the mice injected with ID8-*CCR1*-null cells had developed ascites or other signs of disease at this time point. The median overall survival (OS) for mice injected with ID8-*CCR1*-null cells was 156 days and, therefore, significantly longer compared to both ID8-parental (OS: 64 days) and ID8-CRISPR-Control (OS: 97 days) (Fig. 5e).

The data generated in the ID8 tumor model suggests that deletion of *CCR1* expression resulted in extended survival in vivo. To assess the levels of CCR1 in clinical samples, IHC of a tissue microarray showed that CCR1 was highly expressed in epithelial ovarian cancers across different subtypes, including serous, endometrioid, and clear cell histologies. Interestingly, borderline ovarian tumors, which typically have better prognosis compared to invasive ovarian cancer, did not show any significant expression of CCR1 (Fig. 5g). Based on gene expression data from a cohort of ovarian cancer datasets, including TCGA^29^, patients with high CCR1 expression had a shortened disease-free survival compared to patients with low CCR1 expressing tumors. CCR1 expression levels in stage I and II ovarian cancer patients showed a significant difference in median progression free survival of 37 months with high CCR1 compared to 96 months with low CCR1 expression (HR 2.13; *p* = 0.032) (Fig. 5h, i). These findings lend further support to the important effect of CCR1 on the oncological outcome of ovarian cancer patients.

## Discussion

The current study, to our knowledge, identifies a novel mechanism of ovarian cancer colonization of the omentum mediated by chemokine ligands secreted by omentum resident macrophages. We demonstrate that macrophage secreted CCL6 or CCL23 promotes colonization of ovarian cancer cells to omental milky spots. Targeting of CCR1 in ovarian cancer cells leads to a significant reduction in migration and omental metastasis demonstrating the importance of CCR1 in ovarian cancer cell metastasis.

The role of macrophages or other immune-effector cells in ovarian cancer metastasis is still not well understood. We have previously demonstrated that ovarian cancer cells metastasize initially to milky spots in the omentum but not other intra-abdominal adipose tissues including the mesentery that lack milky spots^21^. This pattern of early omental colonization was independent of the presence of B cells, T cells or NK cells based on studies in immune-deficient mice^21^. Our current study demonstrates that omentum resident macrophages promote early omental metastatic colonization. Gene expression profile of macrophages derived from the omentum demonstrated a tissue-specific pattern compared to other intra-abdominal adipose tissues. Macrophages in other organs including the lung or the liver are likewise characterized by specific gene signatures which convey important and specific functions in their respective tissue microenvironments^30^. Based on our data, omental macrophages secreted chemokine ligands support early metastasis of ovarian cancer cells providing a possible explanation for the frequent involvement of the omentum in ovarian cancer patients.

Chemokine ligands can exert a variety of effects on cellular proliferation, angiogenesis, and metastasis^31,32^. CCL6 and its functional human homolog CCL23 are predominantly expressed by macrophages, eosinophils, and neutrophils^33^. The expression of these chemokine ligands can be upregulated during cancer, peritonitis, and chronic rhinosinusitis^34–36^. CCL6 is a known chemoattractant for macrophages, and to a lesser extent, B cells, T-helper cells, and eosinophils^37,38^. It is able to promote innate immune response by NK cell activation in animal models of peritonitis^39^. IL-4, a known stimulant for an immune-suppressive-M2-macrophage subtype, induces secretion of CCL6 by macrophages^40^. Similarly, CCL23 is IL-4-inducible in a STAT6-dependent manner^41^. In a mouse model of lung cancer, overexpression of CCL6 accelerated tumor growth, and increased metastatic spread^32^. Similarly, higher levels of CCL23 and its corresponding receptor CCR1 correlated with reduced metastasis-free survival of patients with breast cancer^42^.

The omental and intraperitoneal microenvironment, including ascites, contains a variety of chemokine ligands. CCL8 (MCP2), CCL13 (MCP4), CCL14 (HCC1), CCL15 (MIP5), and CCL16 (HCC4) bind to CCR1 albeit with different affinity and could, therefore, potentially promote colonization^35^. However, our experiments in omentum-macrophage-conditioned media underscore the importance of murine CCL6 and human CCL23. Neutralization of CCL6 and CCL23 in these complex environments with a large variety of other chemokine ligands completely abrogated the migration of ovarian cancer cells. We hence propose that the effects in vivo are mainly mediated by CCL6 and CCL23. Conversely, other chemokine receptors may play a role in promoting ovarian cancer cell metastasis to the omentum. CCL6, for example, can bind to CCR3 which has been implicated in metastasis^19^. Neither ID8 nor SKOV3ip.1 cells used in our study expressed CCR3, and we, therefore, excluded its role in the observed reduction of metastasis in *CCR1* deleted cells. However, CCR3 is expressed in human ovarian cancer and might, therefore, be another important receptor to promote metastasis in patients^43^.

A role for CCR1 in cancer metastasis has been suggested in other studies, but not in the context of ovarian cancer or mediated by omental macrophage-derived CCL6/CCL23. CCR1 expression was found to increase in colon cancer cells during metastasis to the liver^44^. In a separate study, CCR1 sustained liver metastasis by promoting local recruitment of bone marrow (BM)-derived cells that secrete the MMP9 and MMP2 metalloproteinases required for tissue invasion^45^. Activation of EGFR signaling via CCR1 has been shown to contribute to breast cancer invasion and metastasis^46^. The CCR1 mediated interaction between macrophages and breast cancer cells induced a chemokine cascade that enhanced metastasis^19^. CCR1 activation enhanced the interaction between “metastasis associated macrophages” (MAM) and cancer cells in part through integrin α4 resulting in increased extravasation of cancer cells and metastasis^47^. In a study by Zhu et al., osteopontin (OPN) was found to upregulate CCR1 expression in hepato-cellular carcinomas. CCR1 knockdown resulted in reduction of migration, invasion and pulmonary metastasis induced by OPN in vitro and in vivo^48^.

CCR1 promotes monocytic tissue infiltration and plays a major role in various other disease processes, including autoimmune diseases like rheumatoid arthritis. Clinical trials targeting CCR1 for the treatment of rheumatoid arthritis and multiple sclerosis have so far shown that the targeting agents used are well tolerated but have limited clinical efficacy^49^. Targeting CCR1 in cancer patients to reduce metastasis or induce changes in the tumor microenvironment might lead to anti-tumor immune responses due to reduction of monocytic infiltration. The findings in our study, and safety and tolerability of CCR1 receptor antagonists in human clinical trials provide a rationale for using CCR1 antagonists as a new therapeutic target in ovarian and other cancers.

Various other cells types, such as neutrophils, mesothelial cells and adipocytes have been described to regulate ovarian cancer metastasis^15,17,50–53^. Neutrophil Extracellular Traps (NET) formation provides a premetastatic omental niche conducive for implantation of ovarian cancer cells. Pharmacological blockade of NET formation decreased omental colonization by ovarian cancer cells^15^. A unique subset of omental tissue-resident macrophages (CD163^+^ Tim4^+^) can promote a premetastatic niche in the omentum^17^. Other studies have focused on the attachment of ovarian cancer cells to mesothelial cells that outline the peritoneal cavity. This process is regulated by multiple adhesion molecules, proteases and extracellular matrix components^50,53^. Fibronectin receptors like α5β1‐integrins, for example, are expressed on the surface of ovarian cancer cells and mediate the adhesion through myosin‐mediated traction forces^54^. Furthermore, TGF‐β secreted by cancer cells activates a RAC1/SMAD‐mediated signaling pathway in mesothelial cells, which results in transcriptional upregulation of the fibronectin gene and induces an EMT‐like phenotype in mesothelial cells^50^. The adhesion of cancer cells to the mesothelium is followed by mesothelial clearance^3,51^. In this process, mesothelial cells are displaced by ovarian cancer spheroids followed by invasion of cancer cells into the deeper layer of peritoneal tissues^54^. Specifically, hypoxic signaling increased expression of lysyl oxidase (LOX) in mesothelial and ovarian cancer cells to promote collagen crosslinking and tumor cell invasion^52^. Further, during ovarian cancer progression, omental adipocytes provide fatty acids as an energy source to ovarian cancer cells^7^.

Based on our current work and the published literature, we propose a multi-step model for omental colonization. Mesothelial cells promote ovarian cancer cell adhesion to the omentum. Subsequent migration and homing to milky spots is induced by omental resident macrophages via chemokine ligands. The interaction between macrophages and cancer cells creates an inflammatory environment that recruits other immune-effector cells, including neutrophils. The formation of NETs further enhances colonization of ovarian cancer cells. Adipocytes then provide the energy required for the continuous growth of tumor cells. Further studies are needed to explore this complex process and identify opportunities for therapeutic interventions.

## Methods

### Cell lines

Human SKOV3.ip1 ovarian cancer cell lines were cultured and maintained at 37 °C in Dulbecco’s Modified Eagle Medium (DMEM) (10-013-CV, Corning Cellgro) supplemented with 10% (v/v) fetal bovine serum (FBS) (S11150, Atlanta Biologicals, Lawrenceville, GA, USA) and 1% penicillin-streptomycin (Pen Strep) (v/v) (15240, Invitrogen). Similarly, the murine ID8 ovarian cancer cell line was cultured in DMEM supplemented with 4% FBS and 1% Penicillin Strep and 1% Insulin-Transferrin-Selenium (25-800-CR, Corning). RAW 264.7 cells and human peripheral blood mononuclear cells (hPBMCs) were cultured in 10% RPMI and 10% FBS and 1% penicillin-streptomycin]. ID8 or SKOV3ip.1 ovarian cancer cells that stably express mRuby (ID8-mRuby/SKOV3ip.1-mRuby) were constructed by lentiviral delivery of pLVX-mRuby expression vector (Stanford genomics facility). The mRuby tagged expression vector along with the packaging vector master mix PMDL(PVSV-G) and PSPAX2 lentiviral expression plasmid was transfected into HEK293T cells to generate the viral conditioned medium. Fluorescence-activated cell sorting using a BD FACS Aria II system (BD Biosciences, San Jose, CA) at the Stanford Flow Cytometry Core Facility was used to select for high mRuby-expressing cells. All cells were maintained under standard tissue culture conditions (i.e., in a humidified incubator at 37 °C supplemented with 5% CO_2_).

### Patient samples

Omenta were obtained from patients under approved IRB protocol at the Dept. of Obstetrics and Gynecology, Stanford Hospital. Informed consent was obtained from each patient prior to sample collection. Omental specimens with HGSC and omenta derived from patients with benign disease were obtained and processed for histological analysis and in vitro assays as described below.

### Mice

All mice were housed, maintained, and euthanized according to APLAC protocol and under the supervision of the Stanford Animal Resource Center. 6–8 weeks, female, inbred C57BL/6 (C57BL/6NHsd; immunocompetent) and nude (Athymic Nude-*Foxn1*nu; T-cell deficient) mice were obtained from Harlan Laboratories (Indianapolis, IN). All animal studies were performed in accordance with Stanford APLAC approved protocols. All mice were randomly assigned to appropriate treatment groups.

#### In vivo experimental metastasis assays

Exponentially growing untagged or mRuby tagged-ID8 parental, ID8-*CCR1*-null cells, ID8-CRISPR-control cells were trypsinized and prepared as a single-cell suspension at a concentration of 2 × 10^6^ cells/mL in ice cold PBS. Animals were injected intraperitoneally with 500 μL of the cell suspension (1 × 10^6^ cells). For all experiments, 500 μL of PBS was injected as a negative control in a parallel group of control mice. At the experimental endpoint (24 h or 7 dpi) of each assay, mice were euthanized in accordance with APLAC protocol via CO_2_ asphyxiation and a secondary method of cervical dislocation was performed. For the time course experiment, grossly visible overt peritoneal metastases that were greater than 1 mm, from each mouse, were counted. Tissues were then harvested, processed, sectioned, and stained as described in “Methods”.

#### Macrophage depletion using clodronate liposomes

For both pre-cancer and post-cancer injection experiments, peritoneal macrophages were depleted by injection of clodronate-containing liposomes (clodronateliposome.org). In vivo depletion of macrophages was assessed for several dosages by cytology performed on omental dissociated cells and by flow cytometry with F4/80 antibody. A dose of 100 μL of liposomes diluted in PBS was selected and administered i.p. to mice every alternate day. Control mice were administered either PBS as a control for unstimulated macrophages or saline liposomes to control for any nonspecific effects of liposome administration. Macrophage depletion was maintained during the experimental period.

### Isolation of murine and human omental macrophages

Omenta excised from C57BL/6 mice (*n* = 9) and processed as three separate groups (*n* = 3 per group). A modified protocol of the adipose tissue dissociation kit (130–105–808, Miltenyi Biotech) was used to obtain a single cell suspension of the mouse omenta. Human omental samples were processed using the human tumor dissociation kit (130-095-929, Miltenyi Biotech) to dissociate soft tumors into a single cell suspension. The cells were ACK lysed and re-suspended in PBS containing 1% FBS for flow cytometry applications to analyze and sort for mouse or human omental macrophages.

### Antibodies and flow cytometry

The isolated mouse and human omental cells were re-suspended in PBS supplemented with 1% FBS. The mouse panel designed to sort for murine omental macrophages included CD45-FITC (clone-30-F11, eBioscience), CD11b-APC (clone-M1/70, eBioscience), F4/80-PE (clone-BM8, eBioscience). Similarly, the human panel designed to sort for human omental macrophages included CD45-FITC (clone-HI30, eBiosciences), CD14-PE (clone-61D3, eBioscience), CD68-PECy7 (Clone-eBioY1/82A, eBioscience). In both the mouse and human flow panel, aqua amine (L34957, Fisher Scientific) was used as the Live/Dead stain and Compensation Beads (01-1111, eBioscience) were used for compensation controls. Labeled cells were sorted by flow cytometry on a BD InfluxFlow Sorter or analysis was performed on BD LSR II at Stanford shared FACS facility. For the RNA-seq analysis, CD45+CD11b+F4/80+ cells were sorted into buffer RLT and were processed immediately for RNA isolation.

### RNA sequencing and analyses

For each sample in the whole transcriptome sequencing library, 43–66 million 75-basepair paired-end reads were acquired from the sequencer. Read quality was determined with FastQC 0.11.4. The reads were aligned to the mouse reference genome (NCBI37/mm9) using STAR 2.4.2a, with splice junctions defined by the GTF file (UCSC). On average, 93% of reads were aligned to the reference genome, and 87% of reads uniquely aligned to the reference genome. Gene expression in raw count was determined by STAR with quant mode, GeneCounts setting. Cufflinks 2.2.1 was used to normalize gene expression in fragments per kilobase per million aligned reads (FPKM). Further, differential expression between the conditions was evaluated using DEseq2 1.12.4. The fold change between each sample is calculated using DEseq2. The genes that were either upregulated 2-fold or downregulated 2-fold were selected for gene set enrichment analysis using DAVID (https://david.ncifcrf.gov/) with gene set annotation GO BP and KEGG database. The top 10 gene sets are only used for visualization.

### CRISPR/Cas9 genome editing

Human (AACTTGTAGTCGATCCAGAA) and murine (GAACACTAGAGAATACAGG) *CCR1* sgRNA sequences were identified using the Broad Institute Genetic Perturbation Platform Web Portal’s sgRNA design tool^55^. Control non-targeting sequences for human (ACGGAGGCTAAGCGTCGCAA) and mouse (GCGAGGTATTCGGCTCCGCG) were taken from the non-targeting sequences in the human and mouse GeCKOv2 libraries^56^. Oligoduplexes were cloned into pLentiCRISPRv2 (a gift from Feng Zhang; Addgene plasmid 52961^56^) and transformed to Stbl3 bacteria. pLentiCRISPRv2 vectors containing the sgRNA guides were transfected with pMD2.G and pSPAX2 (gifts from Didier Trono; Addgene plasmids 12259 and 12260) to 293T cells using Fugene (Roche) in order to produce lentiviral vectors. After 24 h of culture, supernatants were collected, filtered through a 0.45 μm filter and added to monolayers of either ID8 (mouse) or SKOV3ip.1 (human) cell lines. Transduced ID8/SKOV3ip.1 cells were then selected in medium containing 0.6/0.4 µg/mL puromycin for 1 week and individual clones were selected by limiting dilution and then expanded in puromycin containing media.

The targeted genomic regions were amplified by PCR (mouse *CCR1* forward: 5′-TTCCACTGCTTCAGGCTCTT-3′ and reverse: 5′-TCATTTGTTCTGTTCCCCTCTT-3′ and human *CCR1* forward: 5′-TACCAGCCCAAAGAGGTTCA-3′ and reverse: 5′-TGACACGACCACAGAGTTTGA-3′) and analyzed using TIDE-seq^57^. To characterize the specific lesion in each cell line, PCR products were Topo cloned (Life Technologies) and 10 colonies from each isolated cell line was sequenced.

### Cell proliferation assays

For cell proliferation assays, cells were seeded in 96-well plates (1.5 × 10^3^ cells/well). At the indicated time points, cell proliferation was detected by MTS assay (Promega, G3530) according to the manufacturer’s instructions. Data are shown as mean ± s.d. from three independent experiments.

### Real-time RT–PCR

Total RNA was isolated with the RNeasy kit (Qiagen) and reverse-transcribed into cDNA using the Superscript Strand III cDNA SuperMix (Invitrogen). Real-time PCR was performed using SSO Fast Evergreen Supermix (Biorad) on an Applied Biosystems 7500 Fast Real-Time PCR System at Functional Genomics Facility at Stanford. Expression of the target genes were normalized to the housekeeping gene 18S. Mouse RAW 264.7 cells and human PBMC were used as a positive control for the all the gene expression studies.

The target gene-specific primers were obtained from Integrated DNA Technologies (IDT). The primer sequences used in this study are shown in Supplementary Table 2. All real-time RT-PCRs were performed in triplicates and the relative mRNA expression of each target gene was determined by using the formula 2^−ΔCT^ (C_T_, cycle threshold) where ΔC_T_ = C_T_ (target gene) − C_T_ (18S). The comparative expression level of each target gene between different samples was 2^−ΔΔCT^.

### Generation of tissue and cell conditioned media

Three freshly excised mouse omenta were pooled and processed to produce a single cell suspension with minimal cell loss. Gonadal fat was similarly processed in parallel. Macrophages (CD11b^+^ cells) were isolated from stromal and vascular cells (SVC) by positive selection using CD11b microbeads (Miltenyi Biotech) (Supplementary Fig. 2). To assess the functional phenotype, 1 × 10^6^ cells were plated and allowed to condition serum-free media for 24 h after which the media were used in standard transwell migration assay.

### Transwell migration assays

#### Toward chemokines CCL6/CCL23

For cell migration assays, 1.5 × 10^5^ (ID8) or 3 × 10^5^ (SKOV3ip.1) cells in serum-free media were plated in the top chamber of 8.0-μm 12-well plate transwell insert (12-well format, 8-μm pore; BD PharMingen). CCL6 (250-06, Peprotech) CCL23 (300-29, Peprotech), or omental conditioned medium was added to the bottom well of the plate (35-3503, BD Falcon) as chemoattractant. Anti-CCL6 (MAB000487, R&D systems) and anti-CCL23 (MAB371, R&D systems) antibodies were added to omentum-conditioned media in neutralization experiments. For pharmacological Inhibition, CCR1 inhibitor—UCB35625 (2757, Tocris), PI3K inhibitor—BEZ235 (S1009, Selleckchem), MEK inhibitor—trametinib (S2673, Selleckchem) and myosin inhibitor—blebbistatin (1760, Tocris) were used for the migration assays. Analysis was performed on each sample in triplicate. Cells were incubated for a period of 16 h overnight in a 37 °C incubator (5% CO_2_). After incubation, the top and the bottom chambers were washed with PBS and cells were fixed with 4% paraformaldehyde for 10 min. After washing with PBS, the cells were stained with 0.05% Crystal Violet for 40 min. Both the chambers were then washed with tap water and the cells on the top of the insert were gently scraped with a cotton swab. The positive-staining cells adherent to the bottom of the transwell, were examined under the microscope. Data are shown as mean ± s.d. from three independent experiments.

### Protein isolation and western blotting

Protein was extracted from ID8 and SKOV3ip.1 cells treated with CCL6/CCL23 at different time points by RIPA lysis extraction method (pH 7.4) containing Halt protease inhibitor cocktail (#1861278, Thermofisher Scientific) and EDTA (0.5%) under agitation at 4 °C for 30 min. A bicinchoninic acid assay (Pierce, Rockford, IL, USA) was used to determine total protein concentration. Equal amounts of protein were separated on (4–12%) SDS-PAGE and transferred to nitrocellulose membranes (Sigma, St Louis, MO, USA). Membranes were blocked in 5% low-fat dry milk in TBS-T for 1-h at room temperature and then incubated overnight at 4 °C. Dilutions for the primary antibodies were used according to the manufacturer’s recommendation. The primary antibodies used for this study are listed as follows: anti-pERK1/2 (p44/42) (#9101S, Cell Signaling Technologies),anti-total ERK1/2 (#9102S, Cell Signaling Technologies), anti-pAKT (Ser473) (#4058S, Cell Signaling Technologies),anti-total AKT(#9272S, Cell Signaling Technologies), anti-pLIMK (#ab194798,Abcam), anti-p-Cofilin (Ser3) (#3313L, Cell Signaling Technologies), anti-Cofilin (#5175S, Cell Signaling Technologies), anti-alpha tubulin (#3873S, Cell Signaling Technologies). Following day, after three washes for 10 min in TBS-T, the membrane was incubated with IRDye-680 nm conjugated-goat anti-rabbit (#925-32211, LICOR) and IRDye-800nm conjugated-donkey anti-mouse (#926-32212, LICOR) secondary antibodies at 1:5000 for 1-h at room temperature. Membranes were washed for 10 min in TBS-T before being imaged by the LICOR Odyssey Clx imaging system. The intensity of the protein bands was semi-quantified using the Image studio software (Windows application VIO.02), with normalization of each protein against alpha tubulin.

### Phospho-protein array

Protein lysates isolated from ID8 and SKOV3ip.1 cells stimulated with CCL6 or CCL23 at 30 min time points were processed and assayed using the proteome-profiler human phospho-kinase array (#ARY003B, R&D Systems) according to manufactures protocol. Membranes were incubated with protein lysates of ID8 (500 μg) or SKOV3ip.1 (250 μg) for each condition, respectively. The membranes were imaged by Chemiluminescence by using LICOR Odyssey Clx imaging system. Protein bands were semi-quantified using the Image studio software (Windows application VIO.02), using normalization controls as per the manufacture’s protocol.

### Immunohistochemistry

Slides were first deparaffinized with xylene treatment for 60 min, 30 min, and 10 min three times each. Slides were then rehydrated, and antigen retrieval was performed in 1× citrate buffer (pH 6.0) for 5 min in a conventional steamer. Slides were incubated in a blocking buffer solution (Protein block serum-free ready-to-use, DAKO) for 60 min at room temperature. After standard slide preparation as described above, slides were incubated with primary antibody overnight and subsequent secondary antibodies (Supplementary Table 1). After washing, slides were either developed for immunohistochemistry or incubated with DAPI and imaged using an EVOS Fluorescence microscope. Level of CCR1 on patient tissue core samples on a tissue microarray was determined by HALO image analysis software area quantification algorithm (Indica Labs, Corrales, NM).

### Statistics and reproducibility

All grouped data are presented as mean ± s.e.m. Significance between groups was analyzed by one-way ANOVA or Student *t*-test using GraphPad Prism. KM plotter (https://kmplot.com/analysis/) was used to analyze the prognosis of CCR1 in ovarian cancer. The analysis was conducted for all serous samples with an ‘auto-select best cutoff’ feature. The data with statistical analyses were performed with biological replicates on different samples of the same cell type on different days, yielding similar results. The number of biological replicates is included in the figure legends. Human CCL23 ELISA data shown in Fig. 2i is plotted as separate data points to show inter-patient variability.

### Reporting summary

Further information on research design is available in the Nature Research Reporting Summary linked to this article.

## Supplementary information

Supplementary Information

Description of Additional Supplementary Files

Supplementary Data 1

Reporting Summary

## Data Availability

Raw data and analyzed data for the RNA-sequencing and microarray gene expression analysis can be accessed from Gene Expression Omnibus using the accession number GSE153685 and GSE153790, respectively. Source data are available in Supplementary Data 1. All other data are available from authors on reasonable request.

## References

[1] Bray F (2018). Global cancer statistics 2018: GLOBOCAN estimates of incidence and mortality worldwide for 36 cancers in 185 countries. CA Cancer J. Clin..

[2] Siegel RL, Miller KD, Jemal A (2020). Cancer statistics, 2020. CA Cancer J. Clin..

[3] Yeung TL (2015). Cellular and molecular processes in ovarian cancer metastasis. A review in the theme: cell and molecular processes in cancer metastasis. Am. J. Physiol. Cell Physiol..

[4] Lambert AW, Pattabiraman DR, Weinberg RA (2017). Emerging biological principles of metastasis. Cell.

[5] Gerber SA (2006). Preferential attachment of peritoneal tumor metastases to omental immune aggregates and possible role of a unique vascular microenvironment in metastatic survival and growth. Am. J. Pathol..

[6] Pearce OMT (2018). Deconstruction of a metastatic tumor microenvironment reveals a common matrix response in human cancers. Cancer Disco..

[7] Nieman KM (2011). Adipocytes promote ovarian cancer metastasis and provide energy for rapid tumor growth. Nat. Med..

[8] Khan S, Taylor JL, Rinker-Schaeffer CW (2010). Disrupting ovarian cancer metastatic colonization: insights from metastasis suppressor studies. J. Oncol..

[9] Meza-Perez S, Randall TD (2017). Immunological functions of the omentum. Trends Immunol..

[10] Krist LF (1995). Cellular composition of milky spots in the human greater omentum: an immunochemical and ultrastructural study. Anat. Rec..

[11] Liu J, Geng X, Li Y (2016). Milky spots: omental functional units and hotbeds for peritoneal cancer metastasis. Tumour Biol..

[12] Mebius RE (2009). Lymphoid organs for peritoneal cavity immune response: milky spots. Immunity.

[13] Rangel-Moreno J (2009). Omental milky spots develop in the absence of lymphoid tissue-inducer cells and support B and T cell responses to peritoneal antigens. Immunity.

[14] Cleypool, C. G. J., Schurink, B., van der Horst, D. E. M. & Bleys, R. Sympathetic nerve tissue in milky spots of the human greater omentum. *J Anat*. **236**, 156–164 (2019). 10.1111/joa.13077 PMC6904595 31498441

[15] Lee W (2019). Neutrophils facilitate ovarian cancer premetastatic niche formation in the omentum. J. Exp. Med..

[16] Shimotsuma M, Takahashi T, Kawata M, Dux K (1991). Cellular subsets of the milky spots in the human greater omentum. Cell Tissue Res..

[17] Etzerodt, A., et al. Tissue-resident macrophages in omentum promote metastatic spread of ovarian cancer. *J. Exp. Med.* **217**, e20191869 (2020). 10.1084/jem.20191869 PMC7144521 31951251

[18] Linde N (2018). Macrophages orchestrate breast cancer early dissemination and metastasis. Nat. Commun..

[19] Kitamura T (2015). CCL2-induced chemokine cascade promotes breast cancer metastasis by enhancing retention of metastasis-associated macrophages. J. Exp. Med.

[20] Robinson-Smith TM (2007). Macrophages mediate inflammation-enhanced metastasis of ovarian tumors in mice. Cancer Res..

[21] Clark R (2013). Milky spots promote ovarian cancer metastatic colonization of peritoneal adipose in experimental models. Am. J. Pathol..

[22] Orlofsky A, Berger MS, Prystowsky MB (1991). Novel expression pattern of a new member of the MIP-1 family of cytokine-like genes. Cell Regul..

[23] Shay T, Kang J (2013). Immunological Genome Project and systems immunology. Trends Immunol..

[24] Ma B (2004). The C10/CCL6 chemokine and CCR1 play critical roles in the pathogenesis of IL-13-induced inflammation and remodeling. J. Immunol..

[25] Klemke M, Kramer E, Konstandin MH, Wabnitz GH, Samstag Y (2010). An MEK-cofilin signalling module controls migration of human T cells in 3D but not 2D environments. EMBO J..

[26] Makowska KA, Hughes RE, White KJ, Wells CM, Peckham M (2015). Specific myosins control actin organization, cell morphology, and migration in prostate cancer cells. Cell Rep..

[27] Wang W, Eddy R, Condeelis J (2007). The cofilin pathway in breast cancer invasion and metastasis. Nat. Rev. Cancer.

[28] Yamaguchi H, Condeelis J (2007). Regulation of the actin cytoskeleton in cancer cell migration and invasion. Biochim Biophys. Acta.

[29] Gyorffy B, Lanczky A, Szallasi Z (2012). Implementing an online tool for genome-wide validation of survival-associated biomarkers in ovarian-cancer using microarray data from 1287 patients. Endocr. Relat. Cancer.

[30] Gosselin (2014). Environment drives selection and function of enhancers controlling tissue-specific macrophage identities. Cell.

[31] Sarvaiya PJ, Guo D, Ulasov I, Gabikian P, Lesniak MS (2013). Chemokines in tumor progression and metastasis. Oncotarget.

[32] Yi F, Jaffe R, Prochownik EV (2003). The CCL6 chemokine is differentially regulated by c-Myc and L-Myc, and promotes tumorigenesis and metastasis. Cancer Res.

[33] Heng TS, Painter MW, Immunological Genome Project, C. (2008). The Immunological Genome Project: networks of gene expression in immune cells. Nat. Immunol..

[34] LaFleur AM, Lukacs NW, Kunkel SL, Matsukawa A (2004). Role of CC chemokine CCL6/C10 as a monocyte chemoattractant in a murine acute peritonitis. Mediators Inflamm..

[35] Nagarsheth N, Wicha MS, Zou W (2017). Chemokines in the cancer microenvironment and their relevance in cancer immunotherapy. Nat. Rev. Immunol..

[36] Poposki JA (2011). Increased expression of the chemokine CCL23 in eosinophilic chronic rhinosinusitis with nasal polyps. J. Allergy Clin. Immunol..

[37] Asensio VC (1999). C10 is a novel chemokine expressed in experimental inflammatory demyelinating disorders that promotes recruitment of macrophages to the central nervous system. Am. J. Pathol..

[38] Berger MS (1996). The chemokine C10: immunological and functional analysis of the sequence encoded by the novel second exon. Cytokine.

[39] Coelho AL (2007). The chemokine CCL6 promotes innate immunity via immune cell activation and recruitment. J. Immunol..

[40] Orlofsky A, Lin EY, Prystowsky MB (1994). Selective induction of the beta chemokine C10 by IL-4 in mouse macrophages. J. Immunol..

[41] Novak H (2007). CCL23 expression is induced by IL-4 in a STAT6-dependent fashion. J. Immunol..

[42] Yan HH (2015). CCL9 induced by TGFbeta signaling in myeloid cells enhances tumor cell survival in the premetastatic organ. Cancer Res.

[43] Long H (2012). Autocrine CCL5 signaling promotes invasion and migration of CD133+ ovarian cancer stem-like cells via NF-kappaB-mediated MMP-9 upregulation. Stem Cells.

[44] Akram IG, Georges R, Hielscher T, Adwan H, Berger MR (2016). The chemokines CCR1 and CCRL2 have a role in colorectal cancer liver metastasis. Tumour Biol..

[45] Hirai H (2014). CCR1-mediated accumulation of myeloid cells in the liver microenvironment promoting mouse colon cancer metastasis. Clin. Exp. Metastasis.

[46] Shin SY (2017). C-C motif chemokine receptor 1 (CCR1) is a target of the EGF-AKT-mTOR-STAT3 signaling axis in breast cancer cells. Oncotarget.

[47] Qian BZ (2011). CCL2 recruits inflammatory monocytes to facilitate breast-tumour metastasis. Nature.

[48] Zhu Y (2018). C-C chemokine receptor type 1 mediates osteopontin-promoted metastasis in hepatocellular carcinoma. Cancer Sci..

[49] Gladue RP, Brown MF, Zwillich SH (2010). CCR1 antagonists: what have we learned from clinical trials. Curr. Top. Med. Chem..

[50] Kenny HA (2014). Mesothelial cells promote early ovarian cancer metastasis through fibronectin secretion. J. Clin. Invest.

[51] Kenny HA, Kaur S, Coussens LM, Lengyel E (2008). The initial steps of ovarian cancer cell metastasis are mediated by MMP-2 cleavage of vitronectin and fibronectin. J. Clin. Invest..

[52] Natarajan S (2019). Collagen remodeling in the hypoxic tumor-mesothelial niche promotes ovarian cancer metastasis. Cancer Res.

[53] Niedbala MJ, Crickard K, Bernacki RJ (1985). Interactions of human ovarian tumor cells with human mesothelial cells grown on extracellular matrix. An in vitro model system for studying tumor cell adhesion and invasion. Exp. Cell Res.

[54] Iwanicki MP (2011). Ovarian cancer spheroids use myosin-generated force to clear the mesothelium. Cancer Disco..

[55] Doench JG (2016). Optimized sgRNA design to maximize activity and minimize off-target effects of CRISPR-Cas9. Nat. Biotechnol..

[56] Sanjana NE, Shalem O, Zhang F (2014). Improved vectors and genome-wide libraries for CRISPR screening. Nat. Methods.

[57] Brinkman EK, Chen T, Amendola M, van Steensel B (2014). Easy quantitative assessment of genome editing by sequence trace decomposition. Nucleic Acids Res.

