# Supplementary material for: Omental macrophages secrete chemokine ligands that promote ovarian cancer colonization of the omentum via CCR1
Source: Commun Biol. 2020 Sep 22;3:524. doi: 10.1038/s42003-020-01246-z (PMC7508838; doi:10.1038/s42003-020-01246-z)
Supplement: Supplementary file 1 — Supplementary Information [file 42003_2020_1246_MOESM1_ESM.pdf]

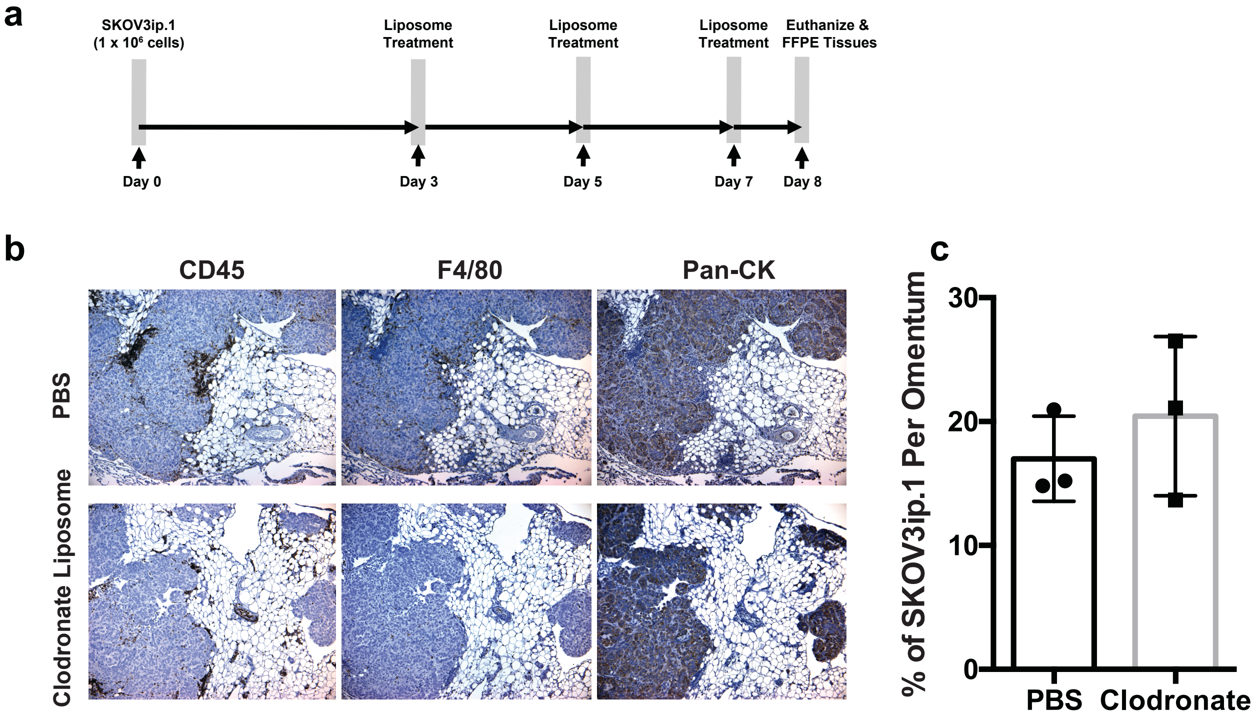

Supplementary Figure 1: Depletion of macrophages following initiation of tumor growth does not reduce omental colonization. (a) Schematic representation of experimental timeline of SKOV3ip.1 cells i.p. injected into athymic nude mice and allowed to metastasize to the omentum for 3 days prior to liposome treatment. (b) Representative omental images of IHC for lymphocytes (CD45), macrophages (F4/80) and cancer cells (pan-CK). (c) Quantification of DAB (pan-CK) staining area (n=5).

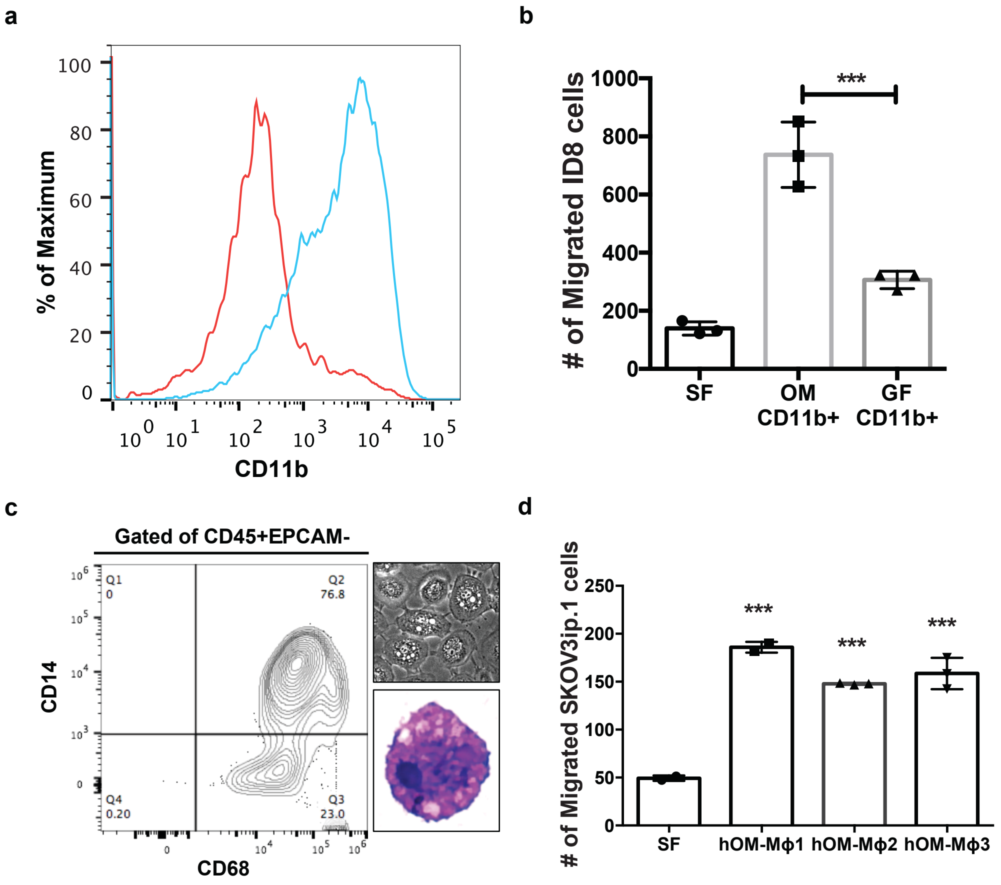

Supplementary Figure 2: Omental macrophage conditioned media promotes in vitro migration of ovarian cancer cells. (a) Cells enriched for CD11b^{+} cells in the Stromal Vascular Fraction (SVF; red line) and the cells eluted from CD11b microbeads (blue line) from C57BL/6 omental (OM). (b) Transwell migration assay of ID8 ovarian cancer cells towards conditioned media derived from omental CD11b+ cells (n=3). (c) (left panel) Macrophages (CD45^{+}CD14^{+}CD68^{+}EPCAM^{-}) purified from human omental tissue by FACS. (right panel) Phase-contrast image of cultured FACS sorted cells and Giemsa stain of cells (cytospin) confirming macrophage-like phenotype. (d) Transwell migration assay of SKOV3ip.1 ovarian cancer cells towards conditioned media derived from human omental macrophages (n=3). Each data point represents an individual patient sample. Statistical significance (***p<0.01) was determined for all conditions versus the serum-free media control by, ordinary one-way ANOVA analysis.

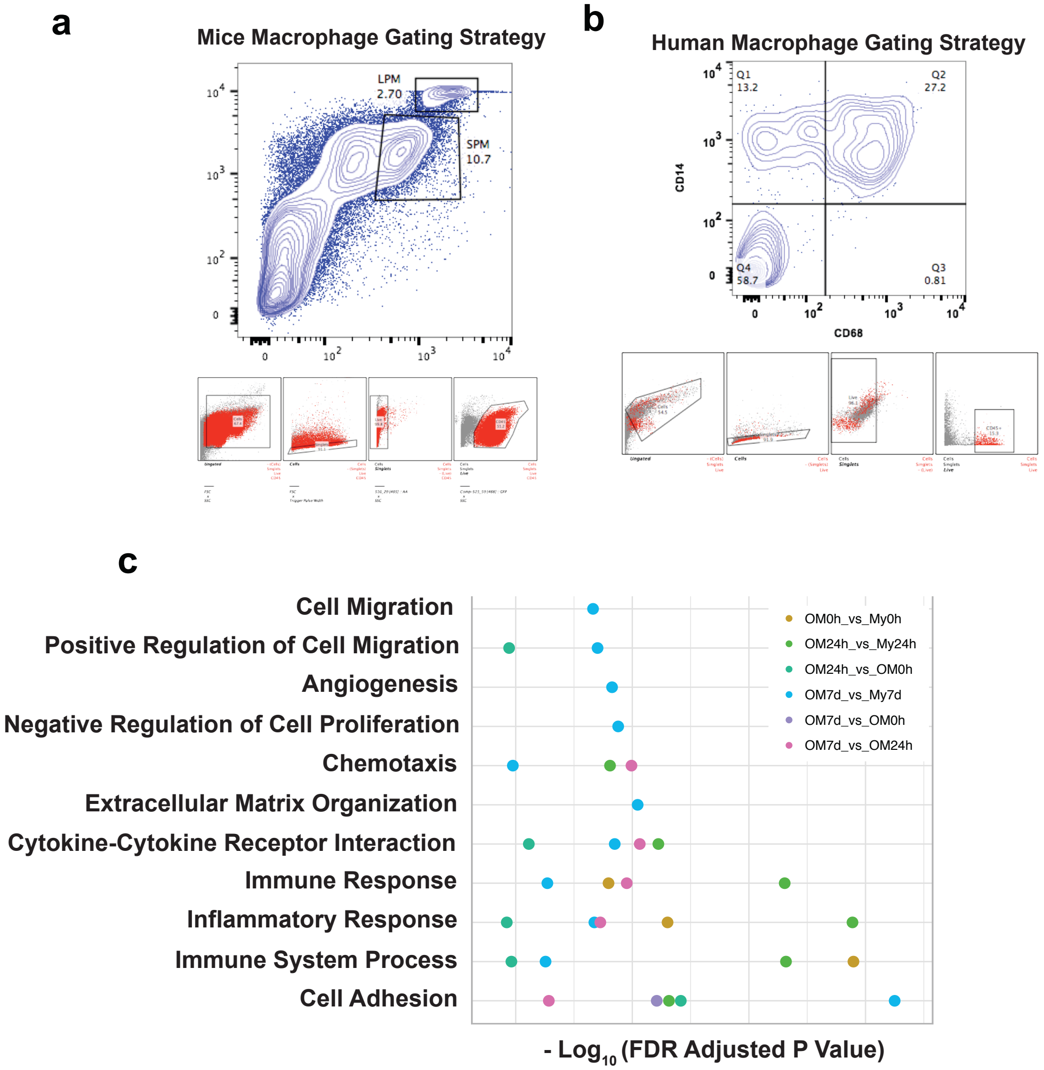

Supplementary Figure 3: Gating strategy used to isolate mouse and human omental macrophages and pathways activated specifically in omental macrophages. (a) Gating strategy used for isolation of mouse omental macrophages ($CD45^+CD11b^+F480^+$) from C57BL/6 omenta (n=9) by flow cytometry. (b) Gating strategy for isolation of human omental macrophages ($CD45^+CD14^+CD68^+$) from human patient samples by flow cytometry. (c) Activated pathways identified from RNA sequencing of mouse omental and mesentery macrophages by gene set enrichment analysis using DAVID (https://david.ncifcrf.gov/). OM=omental macrophages; MY=mesenteric macrophages).

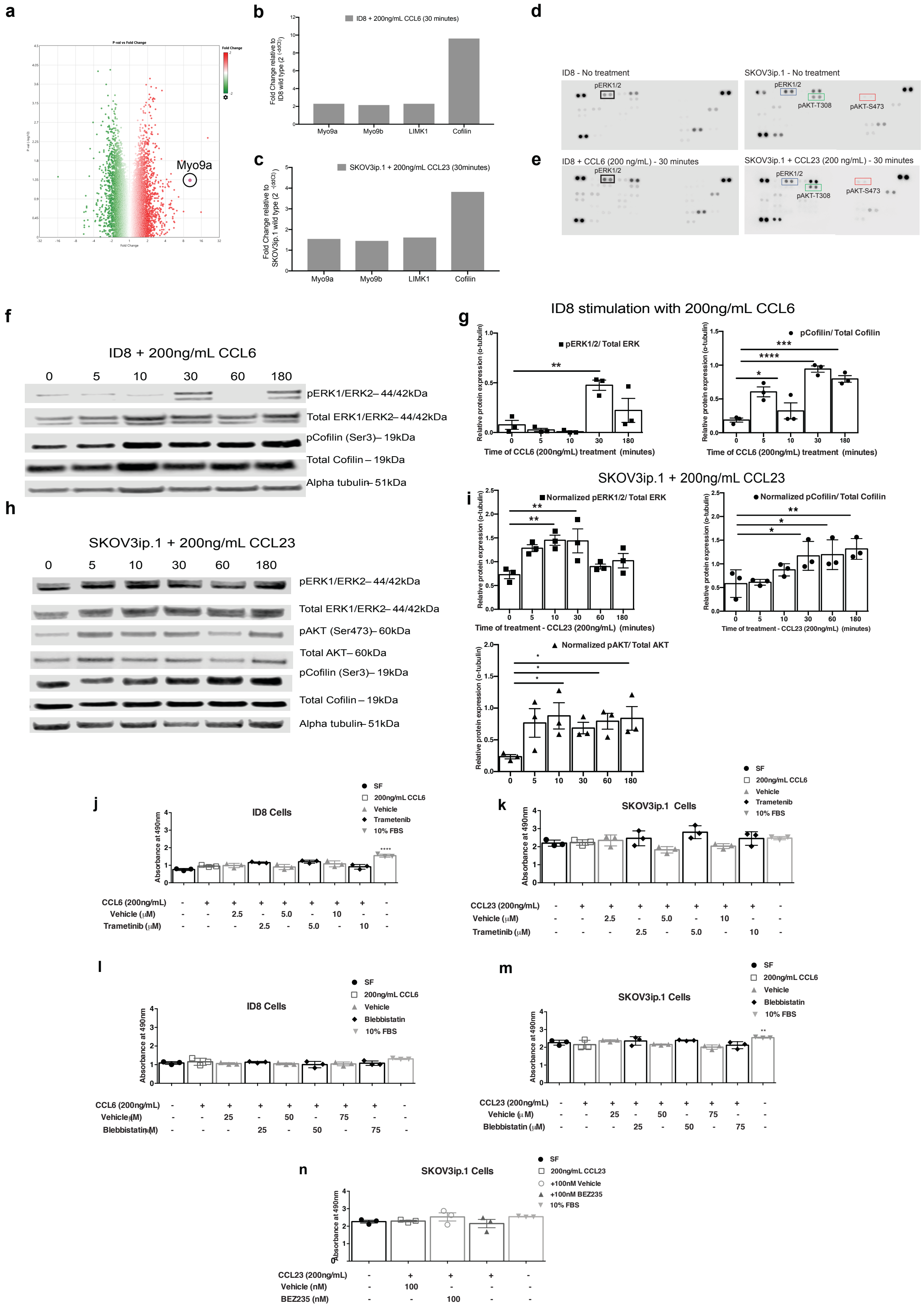
a
Myo9a
b
ID8 + 200ng/mL CCL6 (30 minutes)
Fold Change relative to ID8 wild type (2^-ddCt)
Myo9a
Myo9b
LIMK1
Cofilin
c
SKOV3ip.1 + 200ng/mL CCL23 (30minutes)
Fold Change relative to SKOV3ip.1 wild type (2^-ddCt)
d
ID8 - No treatment
pERK1/2
SKOV3ip.1 - No treatment
pAKT-T308
pAKT-S473
e
ID8 + CCL6 (200 ng/mL) - 30 minutes
SKOV3ip.1 + CCL23 (200 ng/mL) - 30 minutes
f
ID8 + 200ng/mL CCL6
0 5 10 30 60 180
pERK1/ERK2– 44/42kDa
Total ERK1/ERK2– 44/42kDa
pCofilin (Ser3)– 19kDa
Total Cofilin – 19kDa
Alpha tubulin– 51kDa
g
ID8 stimulation with 200ng/mL CCL6
pERK1/2/ Total ERK
pCofilin/ Total Cofilin
Relative protein expression (α-tubulin)
Time of CCL6 (200ng/mL) treatment (minutes)
h
SKOV3ip.1 + 200ng/mL CCL23
0 5 10 30 60 180
pERK1/ERK2– 44/42kDa
Total ERK1/ERK2– 44/42kDa
pAKT (Ser473)– 60kDa
Total AKT– 60kDa
pCofilin (Ser3)– 19kDa
Total Cofilin – 19kDa
Alpha tubulin– 51kDa
i
SKOV3ip.1 + 200ng/mL CCL23
Normalized pERK1/2/ Total ERK
Normalized pCofilin/ Total Cofilin
Normalized pAKT/ Total AKT
Time of treatment - CCL23 (200ng/mL) (minutes)
j
ID8 Cells
SF
200ng/mL CCL6
Vehicle
Trametenib
10% FBS
Absorbance at 490nm
CCL6 (200ng/mL)
Vehicle (μM)
Trametinib (μM)
k
SKOV3ip.1 Cells
CCL23 (200ng/mL)
l
ID8 Cells
Blebbistatin
Blebbistatin (μM)
m
SKOV3ip.1 Cells
n
SKOV3ip.1 Cells
200ng/mL CCL23
+100nM Vehicle
+100nM BEZ235
Vehicle (nM)
BEZ235 (nM)

Supplementary Figure 4: Identification and validation of key pathways activated upon CCR1-CCL6/CCL23 stimulation promoting chemotaxis. (a) Volcano plot showing differentially expressed genes from the Clariom-S-microarray analysis on ID8 stimulated with CCL6 for 6 hours compared to ID8 no treatment cohort (n=3). (b, c) Gene expression analysis by qRT-PCR of MYO9, LIMK1, and Cofilin in ID8 and SKOV3ip.1 cells upon CCL6 or CCL23 stimulation respectively, for 30 minutes. (d, e) Phospho array was performed on ID8 and SKOV3ip.1 cells either unstimulated or stimulated with 200ng/mL CCL6 or CCL23 for 30 minutes. (f, g) Western blot of ID8 cells treated with 200ng/mL CCL6 for 0-180 minutes and its densitometric quantitation. (h, i) Western blot of SKOV3ip.1 cells treated with 200ng/mL CCL23 for 0-180 minutes and its densitometric quantitation. (j, k) Quantitation of cell proliferation using MTS assay of ID8 or SKOV3ip.1 cells in the presence of trametinib and CCL6 or CCL23 (200ng/mL). (l, m) Quantitation of cell proliferation using MTS assay of ID8 or SKOV3ip.1 cells in the presence of blebbistatin and CCL6 or CCL23 (200ng/mL). 10% FBS was used as the positive control. Data plotted represents the mean and s.d of n = 3 replicates per condition. Statistical significance (****$p<0.0001$) was determined by student t test, for the different conditions versus the 200ng/mL CCL6 or CCL23 media control.

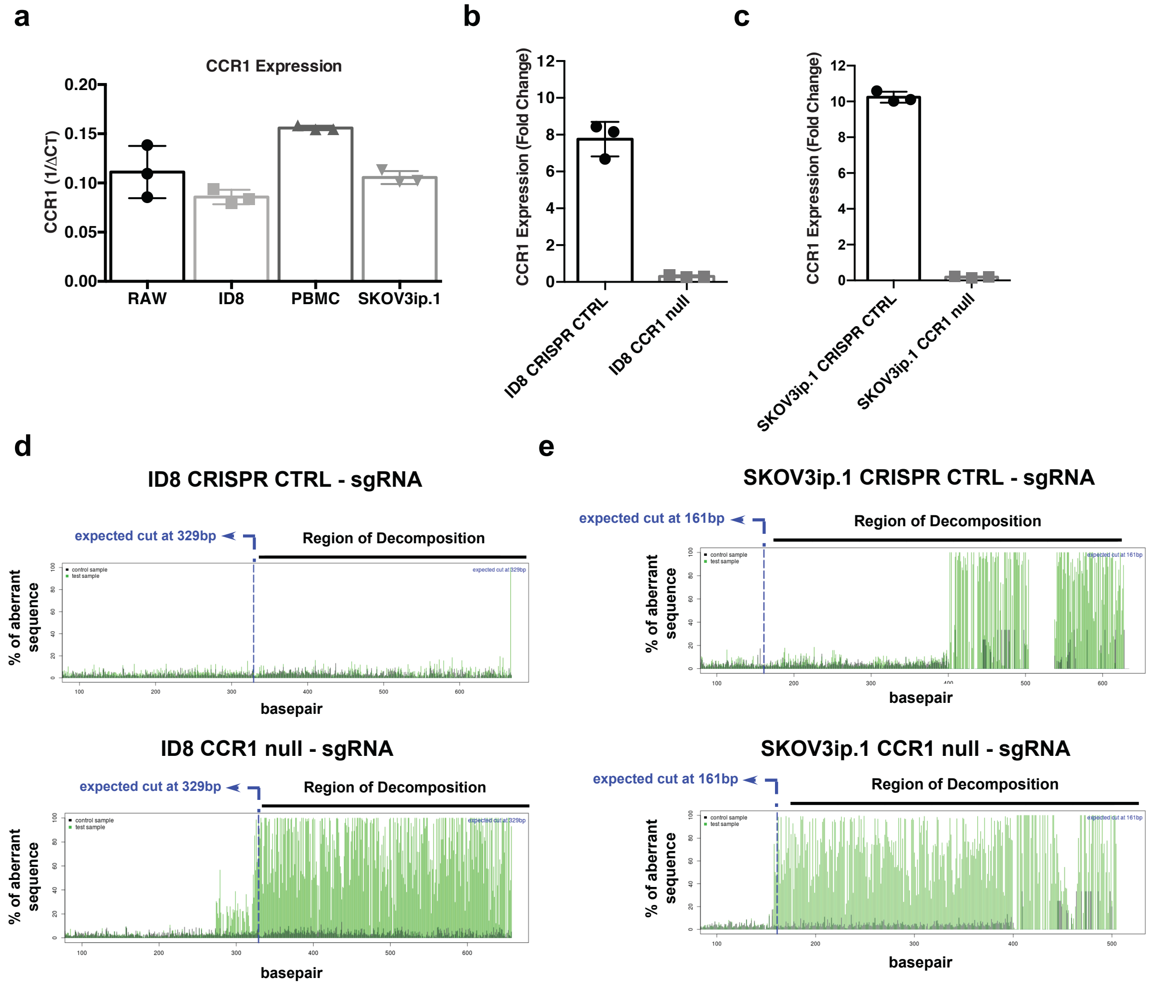

Supplementary Figure 5: Validation of CRISPR mediated CCR1 deletion in ID8 and SKOV3ip cells. (a-c) (a) CCR1 gene is expressed on the ID8/SKOV3ip.1 ovarian cancer cells as confirmed by q-RT PCR. RAW 264.7 and human PBMC were used as the respective positive controls. (b, c) Knockdown of CCR1 gene expression on ID8 and SKOV3ip.1 cells verified by qRT-PCR. (d, e) Sequencing analysis of pLenti CRISPR clones of ID8/SKOV3ip.1 cells confirm genomic deletion of CCR1. (d) Representative TIDE-seq analysis of the ID8-CRISPR-CTRL and ID8-*CCR1*-null clone (green) in comparison to the mouse DNA (black) (decomposition at 329bp; green). (e) Representative TIDE-seq analysis of the SKOV3ip.1-CRISPR-CTRL and SKOV3ip.1-*CCR1*-null clone (green) in comparison to the human DNA (black) (decomposition at 161bp; green). (f, g) Representative quantitation of cell proliferation of ID8 and SKOV3ip.1 parental and CCR1 CRISPR null cells in the presence of CCL6/CCL23 (100ng/mL) by MTS assay.

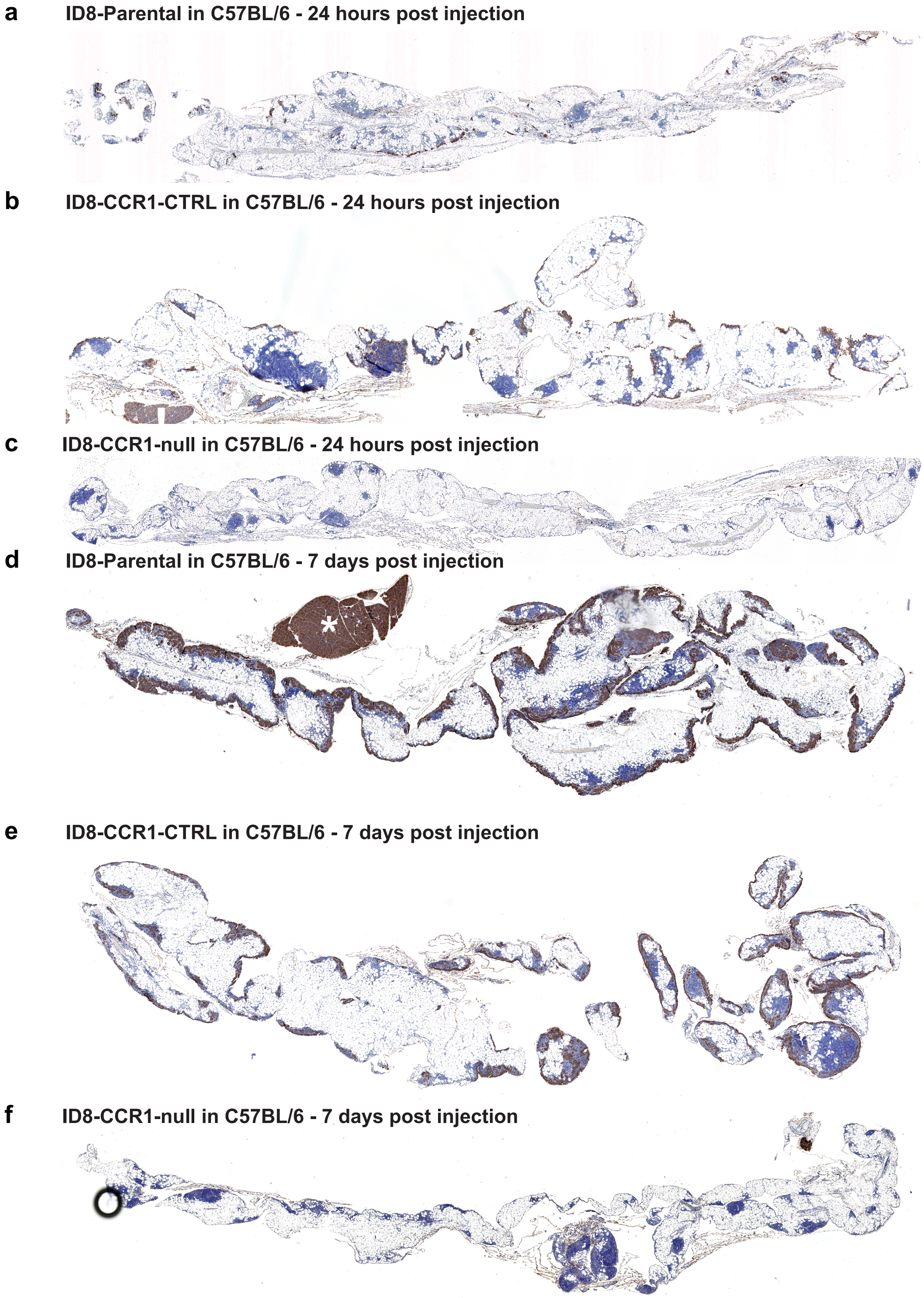
a
ID8-Parental in C57BL/6 - 24 hours post injection
b
ID8-CCR1-CTRL in C57BL/6 - 24 hours post injection
c
ID8-CCR1-null in C57BL/6 - 24 hours post injection
d
ID8-Parental in C57BL/6 - 7 days post injection
*
e
ID8-CCR1-CTRL in C57BL/6 - 7 days post injection
f
ID8-CCR1-null in C57BL/6 - 7 days post injection

Supplementary Figure 6: Omental colonization are significantly reduced in *CCR1* deleted ID8 cells 24 hours and 7 days after tumor initiation. Representative digital scans of whole omental sections collected at specific times after i.p. injection of ID8-parental cells (a, d); ID8-CRISPR-CTRL (b, e) or ID8-*CCR1*-null (c, f) into C57BL/6 mice; ID8 cells detected by IHC for CK8/18 (brown). * indicate non-specific staining of pancreas that were dissected along with the omentum due to its close anatomical proximity in mice peritoneum.
