# Supplementary material for: Omental macrophages secrete chemokine ligands that promote ovarian cancer colonization of the omentum via CCR1
Source: Commun Biol. 2020 Sep 22;3:524. doi: 10.1038/s42003-020-01246-z (PMC7508838; doi:10.1038/s42003-020-01246-z)
Supplement: Supplementary file 2 — Description of Additional Supplementary Files [file 42003_2020_1246_MOESM2_ESM.docx]

**Description of Additional Supplementary Files**

Supplementary Data 1: source data file
