# Supplementary material for: Omental macrophages secrete chemokine ligands that promote ovarian cancer colonization of the omentum via CCR1
Source: Commun Biol. 2020 Sep 22;3:524. doi: 10.1038/s42003-020-01246-z (PMC7508838; doi:10.1038/s42003-020-01246-z)
Supplement: Supplementary file 4 — Reporting Summary [file 42003_2020_1246_MOESM4_ESM.pdf]

nature research

Corresponding author(s): Drs. Venkatesh Krishnan and Oliver Dorigo

Last updated by author(s): 07-03-2020

Reporting Summary

Nature Research wishes to improve the reproducibility of the work that we publish. This form provides structure for consistency and transparency in reporting. For further information on Nature Research policies, see Authors & Referees and the Editorial Policy Checklist.

Statistics

For all statistical analyses, confirm that the following items are present in the figure legend, table legend, main text, or Methods section.

n/a	Confirmed	
☐	☒	The exact sample size (*n*) for each experimental group/condition, given as a discrete number and unit of measurement
☐	☒	A statement on whether measurements were taken from distinct samples or whether the same sample was measured repeatedly
☐	☒	The statistical test(s) used AND whether they are one- or two-sided<br>*Only common tests should be described solely by name; describe more complex techniques in the Methods section.*
☐	☒	A description of all covariates tested
☐	☒	A description of any assumptions or corrections, such as tests of normality and adjustment for multiple comparisons
☐	☒	A full description of the statistical parameters including central tendency (e.g. means) or other basic estimates (e.g. regression coefficient) AND variation (e.g. standard deviation) or associated estimates of uncertainty (e.g. confidence intervals)
☐	☒	For null hypothesis testing, the test statistic (e.g. *F*, *t*, *r*) with confidence intervals, effect sizes, degrees of freedom and *P* value noted<br>*Give P values as exact values whenever suitable.*
☒	☐	For Bayesian analysis, information on the choice of priors and Markov chain Monte Carlo settings
☒	☐	For hierarchical and complex designs, identification of the appropriate level for tests and full reporting of outcomes
☒	☐	Estimates of effect sizes (e.g. Cohen's *d*, Pearson's *r*), indicating how they were calculated

Our web collection on statistics for biologists contains articles on many of the points above.

Software and code

Policy information about availability of computer code

Data collection	Flow cytometry data was collected with BD FACS Diva software<br>Image Studio sofware (LiCOR) was used to capture Western blot images.
Data analysis	Flow cytometry data was analyzed with FlowJo<br>Graphpad Prism was used to analyze data, standard bar graphs, and to perform statistical analysis.<br>CRISPR/Cas9 deletion was confirmed by TIDE-seq<br>HALO image analysis software was used to quantitate CCR1 expression level on patient tissue microarray

For manuscripts utilizing custom algorithms or software that are central to the research but not yet described in published literature, software must be made available to editors/reviewers. We strongly encourage code deposition in a community repository (e.g. GitHub). See the Nature Research guidelines for submitting code & software for further information.

Data

Policy information about availability of data

All manuscripts must include a data availability statement. This statement should provide the following information, where applicable:

- Accession codes, unique identifiers, or web links for publicly available datasets
- A list of figures that have associated raw data
- A description of any restrictions on data availability

RNA-sequencing data can be accessed from Gene Expression Omnibus using the accession number GSE153685 and the microarray data using accession number GSE153790.
All other source data related to this manuscript is available upon reasonable request from the corresponding author.

Field-specific reporting

Please select the one below that is the best fit for your research. If you are not sure, read the appropriate sections before making your selection.

☒ Life sciences ☐ Behavioural & social sciences ☐ Ecological, evolutionary & environmental sciences

For a reference copy of the document with all sections, see nature.com/documents/nr-reporting-summary-flat.pdf

Life sciences study design

All studies must disclose on these points even when the disclosure is negative.

Sample size	For all in vivo and in vitro studies, biological measurements, at least 3 biological replicates were used, which allowed a fold change of 0.5 to be calculated as significant ($p<0.05$; Type I error = 0.05; power 90%). Specifically, for the RNA-sequencing, omenta excised from C57BL/6 mice (n=9) and processed as three separate groups (3 omenta were grouped per tube making up an n of 1; RNA-seq was carried out on n of 3).
Data exclusions	No data were excluded
Replication	For the in vivo follow up experiments except the RNA-seq experiment was carried out two independent times. All experiments were reproduced to reliably support conclusions stated in the manuscript.
Randomization	Animals were randomly divided into experimental groups.
Blinding	N/A

Reporting for specific materials, systems and methods

We require information from authors about some types of materials, experimental systems and methods used in many studies. Here, indicate whether each material, system or method listed is relevant to your study. If you are not sure if a list item applies to your research, read the appropriate section before selecting a response.

Materials & experimental systems

n/a	Involved in the study	
☐	☒	Antibodies
☐	☒	Eukaryotic cell lines
☒	☐	Palaeontology
☐	☒	Animals and other organisms
☒	☐	Human research participants
☒	☐	Clinical data

Methods

n/a	Involved in the study	
☒	☐	ChIP-seq
☐	☒	Flow cytometry
☒	☐	MRI-based neuroimaging

Antibodies

Antibodies used	anti-pERK1/2 (p44/42) (#9101S, Cell Signaling Technologies),anti-total ERK1/2 (#9102S, Cell Signaling Technologies), anti-pAKT (Ser473) (4058S, Cell Signaling Technologies),anti-total AKT(9272S, Cell Signaling Technologies), anti-pLIMK (ab194798,Abcam), anti-p-Cofilin (Ser3) (3313L, Cell Signaling Technologies), anti-Cofilin (5175S, Cell Signaling Technologies), anti-alpha tubulin (3873S, Cell Signaling Technologies), IRDye -680nm conjugated-goat anti-rabbit (925-32211, LICOR) and IRDye-800nm conjugated-donkey anti-mouse (926-32212, LICOR). CD45-FITC (clone-30-F11, eBioscience), CD11b-APC (clone-M1/70, eBioscience), F4/80-PE (clone-BM8, eBioscience), CD45-FITC (clone-HI30, eBiosciences), CD14-PE (clone-61D3, eBioscience), CD68-PECy7 (Clone-eBioY1/82A, eBioscience), aqua amine (L34957, Fisher scientific), Compensation Beads (01-1111, eBioscience). CCL6 (mybioscience, MBS2529419), F4/80 (Biorad, MCA497GA), CCL23 (Atlas Antibodies, HPA042015), CD68 (Thermo Scientific, MA5-13324), CCR1 (R&D Systems, AF5986), TROMA (krt8) (DHSB), hCCR1 (SinoBiological, 100449-T46), EpCAM (ThermoFisher, MA5-12153). Anti-CCL6 (MAB000487, R&D systems) and anti-CCL23 (MAB371, R&D systems)
Validation	Specificity of each antibody was validated by the manufacturer and data in the manuscript supports their specificity.

Eukaryotic cell lines

Policy information about cell lines

Cell line source(s)	The SKOV3ip.1 human ovarian carcinoma cell line was generously supplied by Dr. Gordon Mills (MD Anderson Cancer Center, Houston, TX). The ID8 mouse ovarian carcinoma cell line, derived from and syngeneic to mice of the C57BL/6 background, was generously provided by Dr. Katherine Roby (University of Kansas Medical Center, Kansas City, KS)
Authentication	Cell lines were authenticated by IDEXX BioAnalytics. Detailed report will be provided upon request.

Mycoplasma contamination	All cell lines used in this study was tested by IDEXX BioAnalytics and test results were negative for mycoplasma.
Commonly misidentified lines (See ICLAC register)	None

Animals and other organisms

Policy information about studies involving animals; ARRIVE guidelines recommended for reporting animal research

Laboratory animals	6-8 weeks, female, inbred C57BL/6 (C57BL/6NHsd; immunocompetent) and nude (Athymic Nude-Foxn1nu; T-cell deficient) mice were obtained from Harlan Laboratories (Indianapolis, IN).
Wild animals	N/A
Field-collected samples	N/A
Ethics oversight	All animal studies were performed in accordance with Stanford APLAC approved protocols.

Note that full information on the approval of the study protocol must also be provided in the manuscript.

Flow Cytometry

Plots

Confirm that:

☒ The axis labels state the marker and fluorochrome used (e.g. CD4-FITC).

☒ The axis scales are clearly visible. Include numbers along axes only for bottom left plot of group (a 'group' is an analysis of identical markers).

☒ All plots are contour plots with outliers or pseudocolor plots.

☒ A numerical value for number of cells or percentage (with statistics) is provided.

Methodology

Sample preparation	The isolated mouse and human omental cells were re-suspended in PBS supplemented with 1% FBS. In both the mouse and human flow panel, aqua amine (L34957, Fisher scientific) was used as the Live/Dead stain and Compensation Beads (01-1111, eBioscience) were used for compensation controls.
Instrument	Labeled cells were sorted by flow cytometry on a BD InfluxFlow Sorter or analysis was performed on BD LSR II at Stanford shared FACS facility.
Software	Flow cytometry data was collected with BD FACS Diva software and analyzed with FlowJo
Cell population abundance	For the RNA-seq analysis, CD45+CD11b+F4/80+ cells were sorted into buffer RLT and were processed immediately for RNA isolation. Labeled cells were sorted by flow cytometry on a BD InfluxFlow Sorter
Gating strategy	Detailed gating strategy of mice and human omental macrophages are listed in Supplementary Figure 3A and B: Forward scatter vs. side scatter plot with gate 1 to separate singlets and gate 2 for cell events from debris and live-CD45 cells.

☒ Tick this box to confirm that a figure exemplifying the gating strategy is provided in the Supplementary Information.
